# Improvement of the catalytic activity and thermostability of a hyperthermostable endoglucanase by optimizing N-glycosylation sites

**DOI:** 10.1186/s13068-020-1668-4

**Published:** 2020-02-26

**Authors:** Chao Han, Qunqing Wang, Yanxu Sun, Ruirui Yang, Mengyu Liu, Siqi Wang, Yifan Liu, Lifan Zhou, Duochuan Li

**Affiliations:** Shandong Key Laboratory for Agricultural Microbiology, College of Plant Protection, Shandong Agricultural University, Tai’an, 271018 Shandong China

**Keywords:** Endoglucanase, N-glycosylation site, Thermostability, Specific activity

## Abstract

**Background:**

Endoglucanase has been extensively employed in industrial processes as a key biocatalyst for lignocellulosic biomass degradation. Thermostable endoglucanases with high catalytic activity at elevated temperatures are preferred in industrial use. To improve the activity and thermostability, site-directed mutagenesis was conducted to modify the N-glycosylation sites of the thermostable β-1,4-endoglucanase CTendo45 from *Chaetomium thermophilum*.

**Results:**

In this study, structure-based rational design was performed based on the modification of N-glycosylation sites in CTendo45. Eight single mutants and one double mutant were constructed and successfully expressed in *Pichia pastoris*. When the unique N-glycosylation site of N88 was eliminated, a T90A variant was active, and its specific activity towards CMC-Na and β-d-glucan was increased 1.85- and 1.64-fold, respectively. The mutant R67S with an additional N-glycosylation site of N65 showed a distinct enhancement in catalytic efficiency. Moreover, T90A and R67S were endowed with extraordinary heat endurance after 200 min of incubation at different temperatures ranging from 30 to 90 °C. Likewise, the half-lives (*t*_1/2_) indicated that T90A and R67S exhibited improved enzyme thermostability at 80 °C and 90 °C. Notably, the double-mutant T90A/R67S possessed better hydrolysis activity and thermal stability than its single-mutant counterparts and the wild type.

**Conclusions:**

This study provides initial insight into the biochemical function of N-glycosylation in thermostable endoglucanases. Moreover, the design approach to the optimization of N-glycosylation sites presents an effective and feasible strategy to improve enzymatic activity and thermostability.

## Background

Plant polysaccharide depolymerization by synergistic enzyme cocktails is crucial for the production of lignocellulosic biofuels [1]. These biomass-degrading enzymes are primarily composed of a diverse set of glycoside hydrolases (GHs) that efficiently catalyze the conversion of lignocellulose to glucose [2]. Endoglucanase (EC 3.2.1.4), which randomly deconstructs the internal β-1,4-glucosidic linkages in amorphous regions of biopolymer fibers to trigger an initial catalytic attack on cellulose chains, is an essential biocatalyst for cellulose degradation [3].

Since elevating temperature generally improves catalytic efficiency and simultaneously reduces microbial contamination, thermostability is a desired quality for endoglucanases in practice [4, 5]. Additionally, utilizing potent thermostable endoglucanases with optimal activity at high temperatures can accelerate the hydrolysis process, shorten the reaction period, and enhance cost competitiveness [6, 7]. Consequently, the enhancement of advantageous characteristics is desirable to generate superior endoglucanases [8]. Rational engineering coupled with structural analysis and functional prediction is an efficient genetic approach to optimize enzyme properties [9, 10]. To date, many thermostable endoglucanases from diverse origins and GH families have been engineered to improve their specific activity and thermal stability [11–14].

N-glycosylation is a ubiquitous posttranslational modification involving the covalent attachment of a carbohydrate unit at the asparagine residue within the sequon Asn-Xaa-Ser/Thr (where Xaa cannot be Pro) [15]. Cellulases secreted from filamentous fungi are often decorated with N-glycosylation, which plays varied roles in myriad biological functions [16]. For example, the modification of N-glycans in GH6 and GH7 family cellobiohydrolases can improve their biochemical properties in some cases, including thermal and proteolytic stability, hydrolytic activity, and substrate binding [16–20]. In addition, N-glycosylation affects the proper folding, enzymatic characteristics, and production of fungal GH1 and GH3 family β-glucosidases [21, 22]. However, the underlying role of N-glycosylation in the enzymatic activity and stability of endoglucanases is rarely reported [16], especially for industrially important thermostable endoglucanases. Furthermore, ideal engineering of thermostable endoglucanases should be conducted, along with modification of N-glycosylation sites, to generate mutants with optimized performance.

In our previous study, a novel thermostable β-1,4-endoglucanase, CTendo45, which is a typical GH45 family member, was identified in the thermophilic fungus *Chaetomium thermophilum* [12, 23, 24]. In this study, rational engineering was performed to further improve the specific activity and thermostability of CTendo45 by removing the existing N-glycosylation sites and introducing additional ones using site-directed mutagenesis, providing a feasible pathway for improved enzyme redesign proposals.

## Results and discussion

### Structural characterization

**Fig. 1:**
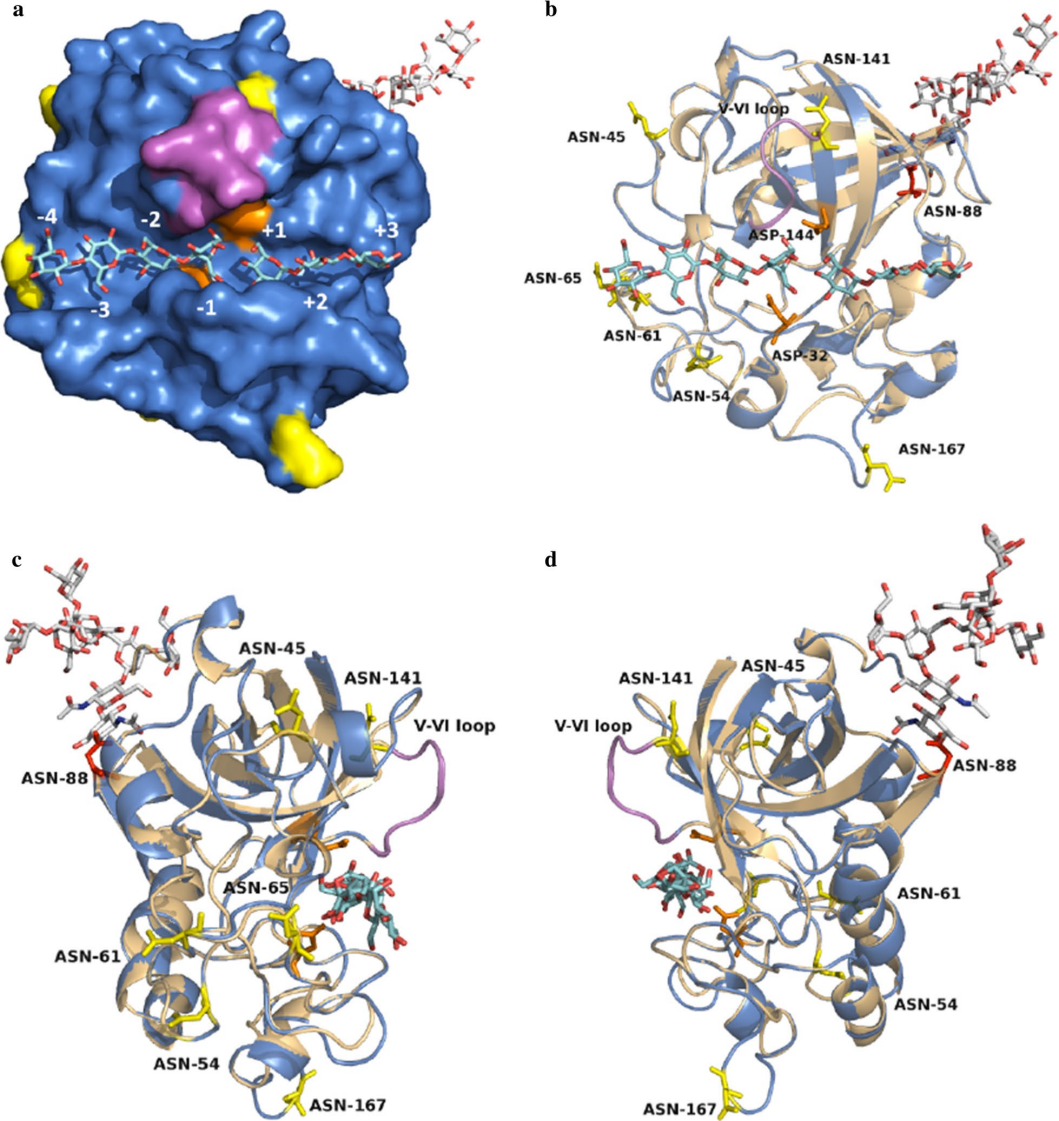
Homologous modeling of CTendo45 using the *Thielavia terrestris* β-1,4-endoglucanase TtCel45A (PDB: 5GLY) as a template. **a** The solvent accessible surface. Cellotriose and cellotetraose molecules, bound in the substrate-binding cleft, are shown in cyan. The branched N-glycan attached to the residue Asn88 in CTendo45 is represented as a white and red stick. The V–VI loop is noted in purple. Catalytic residues and the additional histidine residues are noted in orange and yellow, respectively. The intrinsic histidine residue was noted in red. **b** The 3D superposition between CTendo45 (marine) and TtCel45A (wheat). The spatial positions of valuable residues used in this study are marked by colored sticks. A front view of substrate-binding cleft. **c** Rotating the configuration 90° anti-clockwise. **d** Rotating 90° clockwise. All of the structural diagrams were drawn using PyMOL software

Homologous modeling is an efficient and general approach to predict the three‐dimensional (3D) structure of structurally uncharacterized proteins [25, 26]. Although the 3D structure of CTendo45 has not been solved, crystal structures of some homologous GH45 endoglucanases are available from different organisms [27–30]. Among them, the *Thielavia terrestris* β-1,4-endoglucanase TtCel45A (PDB: 5GLY), which is a typical GH45 endoglucanase and shares the highest amino acid identity (64%) with CTendo45, was used as a basis to construct the protein model for structural characterization [28]. The root-mean-square deviation (RMSD) value and the global model quality estimation (GMQE) score used for model quality evaluation are 0.095 Å and 0.8, respectively, indicating that the model is reliable for homologous structural analysis [31, 32]. As shown in Fig. 1, CTendo45 possesses a six-stranded β-barrel framework and a characteristic region with several interconnecting loops. These two main portions are separated by a substrate-binding cleft, which contains the catalytic center (subsites − 4 to + 3) [28]. The latter comprises Asp144 (catalytic acid) and Asp32, the catalytic base that confers nucleophilic enhanced character to the catalytic water [29, 33, 34]. Residue Asn88, the unique N-glycosylation site in CTendo45 (Additional file 1: Table S1), lies at the β-barrel domain on the opposite side of the catalytic domain and is decorated with a branched oligosaccharide chain (Fig. 1).

**Fig. 2:**
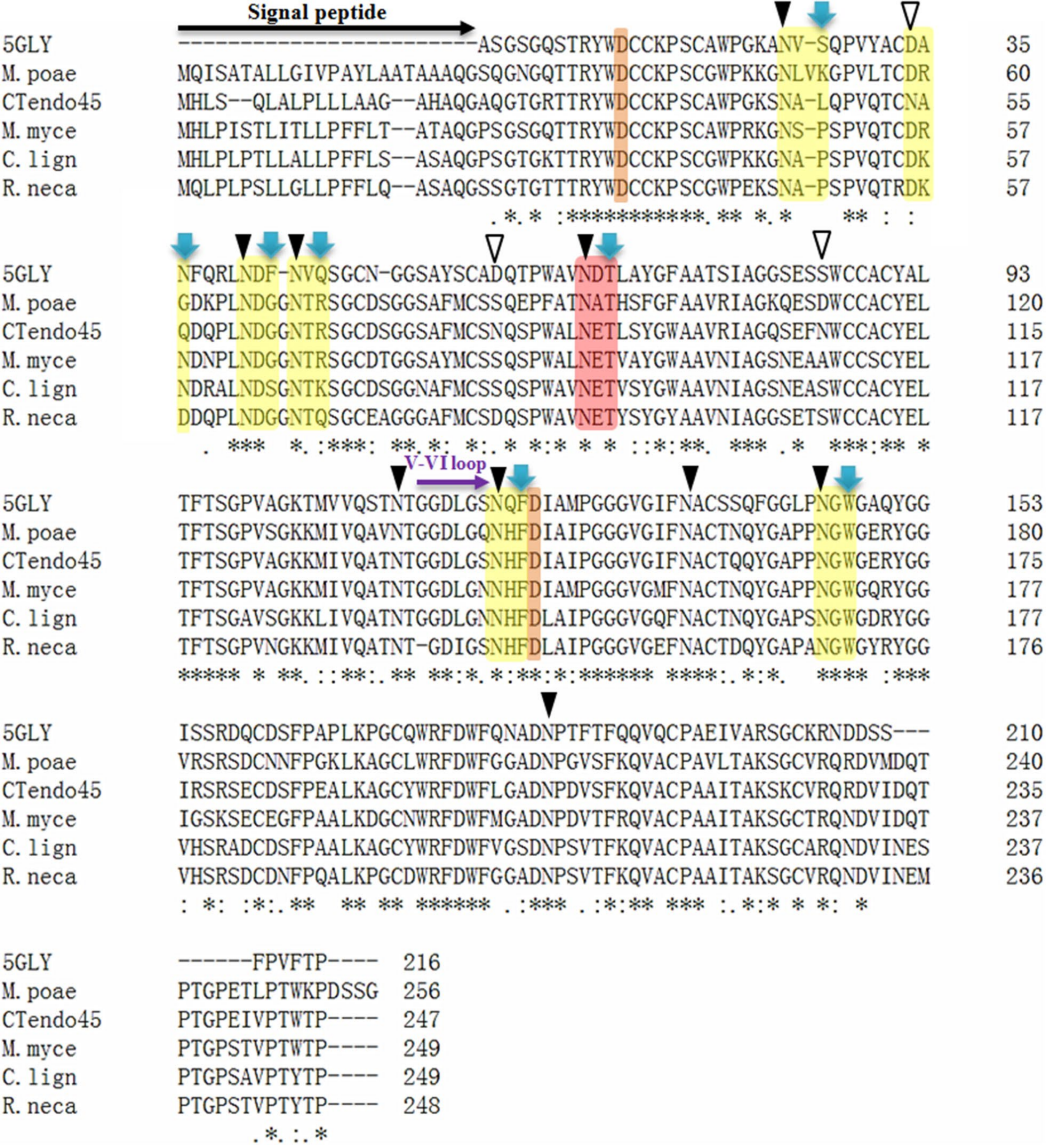
Sequence alignment of CTendo45 with other homologous GH45 endoglucanases using Clustal Omega. These enzymes are isolated from *Thielavia terrestris* (PDB: 5GLY, 64% of sequence identity), *Magnaporthiopsis poae* ATCC 64411 (KLU88048, 77% of sequence identity), *Madurella mycetomatis* (KXX82926, 78% of sequence identity), *Coniochaeta ligniaria* NRRL 30616 (OIW24112, 78% of sequence identity), and *Rosellinia necatrix* (GAP84246, 72% of sequence identity), respectively. Asterisk indicates the positions which have a single, fully conserved residue. Colon indicates the strongly similar parts among homologous sequences and period means the weakly similar parts among homologous sequences. The potential signal peptide is signed with a black arrow and the V–VI loop is signed with a purple arrow. Conserved and nonconserved asparagine residues in CTendo45 are noted by closed and open inverted triangles, respectively. Highlight blocks specify the catalytic residues Asp32 and Asp144 of CTendo45 in orange. The modifiable and intrinsic N-glycosylation sequences are shaded in yellow and red, respectively. All mutant sites in this study are noted by blue vertical arrows

The multiple sequence alignment profile shows nine conserved and three nonconserved Asn residues (Fig. 2). Combining this information with protein structure analysis reveals that only five conserved Asn residues and one nonconserved Asn residue (N45, N61, N65, N141, N167, and N54, respectively) actually form part of the potential N-glycosylation sequon (Asn-Xaa-Ser/Thr, where Xaa ≠ Pro) after appropriate modifications by site-directed mutagenesis [35] (Figs. 1, 2). To create the N-glycosylation sequons, L47, Q56, G63, R67, T90, F143, and W169 were replaced by a Thr or Ser residue (Additional file 1: Figs. S1, S2), generating mutants L47T, Q56T, G63T, R67S, T90A, F143T, F143S, and W169S, respectively (Table 1). Moreover, the double-mutant T90A/R675S was created using the genetic background of each single mutant to further improve the hydrolytic activity and thermostability.

**Table 1:** N-glycosylation motifs of CTendo45 and its mutants

Enzyme	Motif	Modification	Existing sites
CTendo45	N88-E89-T90	–	N88
L47T	N45-A46-T47	Addition	N45 and N88
Q56T	N54-A55-T56	Addition	N54 and N88
G63T	N61-D62-T63	Addition	N61 and N88
R67S	N65-T66-S67	Addition	N65 and N88
T90A	N88-E89-A90	Deletion	–
F143T	N141-H142-T143	Addition	N141 and N88
F143S	N141-H142-S143	Addition	N141 and N88
W169S	N167-G168-S169	Addition	N167 and N88
R67S/T90A	N65-T66-S67	Substitution	N65
	N88-E89-A90		

Glycans linked to the marked Asn residues were modified in mutants

### Production and purification of mutant enzymes

**Fig. 3:**
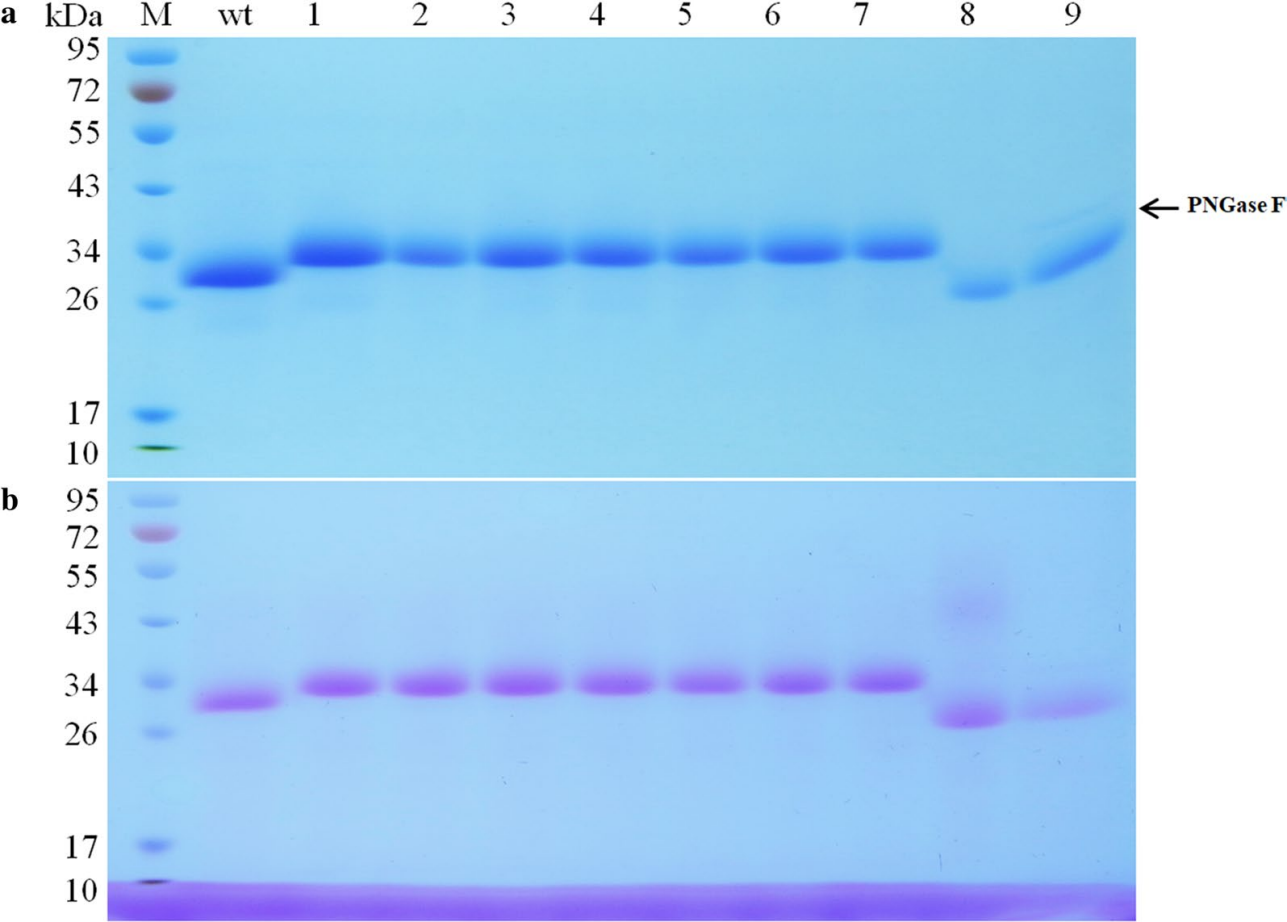
SDS-PAGE analysis of purified recombinant enzymes. Lane M, molecular mass markers; lane wt, the wild-type CTendo45; lane 1–7, L47T, Q56T, G63T, R67S, F143T, F143S and W169S; lane 8, T90A; lane 9, the purified CTendo45 treated with PNGase F. **a** Coomassie blue staining. **b** Carbohydrate staining

To determine the enzymatic characteristics, CTendo45 and its mutants were heterologously expressed in *P. pastoris* and purified using Ni^2+^ affinity chromatography. The similar protein yields implied that the attachment of N-glycans to different sites has hardly any influence on efficient enzyme expression (Additional file 1: Table S2). SDS-PAGE analysis showed that each single mutant with an additional N-glycosylation site presented as a single band at approximately 34 kDa, which is higher than that of the wild type (approximately 32 kDa) (Fig. 3a) because of the artificial attachment of oligosaccharides under the same culture conditions [36]. After the deglycosylation of N-linked glycans with PNGase F, the enzyme molecular mass decreased to 28 kDa, which was consistent with the size of the N-deglycosylated mutant T90A. The molecular mass of the double-mutant R67S/T90A is nearly the same as that of the wild type (Additional file 1: Fig. S3). Glycoprotein staining further confirmed that residue N88 was the unique N-glycosylation site in CTendo45. Moreover, this result indicated that the N-glycan was successfully attached to each additional mutation as a single clear band (Fig. 3b). In particular, it is worth mentioning that the bands of T90A and deglycosylated wild-type also appeared in glycoprotein staining, which resulted from O-linked glycans, as there are three predicted *O*-glycosylation sites (T52, T235, and T237) in CTendo45. The mass spectrum of the tryptic digest of CTendo45 and its mutants further confirmed the addition or deletion of N-glycan in a specific position (Additional file 1: Table S3 and Fig. S4).

### Effect of N-glycosylation on activity

**Fig. 4:**
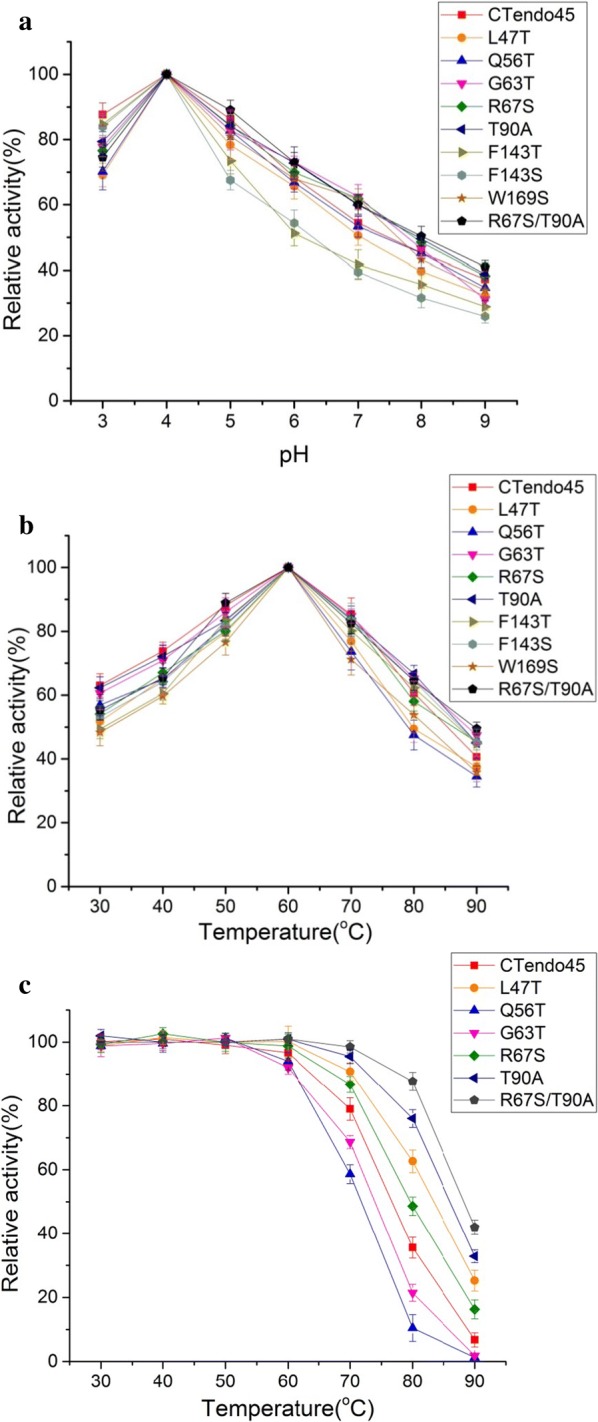
Enzymatic properties of wild-type CTendo45 and its mutants against CMC-Na. **a** Optimal reaction pH. **b** Optimal reaction temperature. **c** Thermostability

The optimal pH values displayed no obvious differences, with relatively high catalytic activity against CMC-Na under acidic conditions (pH 4) (Fig. 4a). In addition, all mutants had similar temperature optima to that of wild-type CTendo45 at 60 °C (Fig. 4b). These results were consistent with that of the optimum activity assay using the native substrate of β-d-glucan (Additional file 1: Fig. S5), which is likely attributable to the lack of apparent conformational rearrangements as a result of changes in N-glycosylation sites [37].

Compared with the wild type, the mutant T90A, eliminating the unique N88-glycosylation site, showed enhanced hydrolytic activity towards CMC-Na and β-d-glucan with increases of 1.85- and 1.64-fold, respectively (Table 2). The intrinsic glycosylation with the branched glycan attached to residue N88 acts a fastening clamp that restrains the conformation of the backbone β-barrel domain and further maintains a relatively crowded and confined structure for the whole protein, especially for the substrate-binding cleft [38]. The absence of the glycan, therefore, initiates a moderate loosening of the buried cleft, leading to functional improvement of the catalytic residues [16]. In addition, a distinct enhancement of hydrolytic performance was noted for the mutant R67S (Table 2), probably attributable to the dynamic interaction between cellulose chains and the additional oligosaccharide attached at residue N65, which is located close to the cleft (Fig. 1) [17, 39]. Based on these observations, the double-mutant R67S/T90A was generated. The activity of R67S/T90A was increased and exhibited maxima of 2.26- and 1.94-fold against CMC-Na and β-d-glucan, respectively (Table 2).

**Table 2:** Activities of CTendo45 and its mutants on CMC-Na and β-d-glucan as substrates

Substrate	Enzyme	Activity (IU/mg)	Fold
CMC-Na	CTendo45	1.21 ± 0.08	1.00
	L47T	1.36 ± 0.06	1.12
	Q56T	1.28 ± 0.10	1.06
	G63T	0.91 ± 0.08	0.75
	R67S	1.58 ± 0.12	1.31
	T90A	2.24 ± 0.18	1.85
	F143T	0.20 ± 0.04	0.17
	F143S	0.16 ± 0.02	0.13
	W169S	0.07 ± 0.01	0.06
	R67S/T90A	2.73 ± 0.15	2.26
β-d-glucan	CTendo45	2.07 ± 0.14	1.00
	L47T	2.17 ± 0.08	1.05
	Q56T	2.09 ± 0.15	1.01
	G63T	1.71 ± 0.11	0.83
	R67S	2.62 ± 0.13	1.27
	T90A	3.39 ± 0.21	1.64
	F143T	0.98 ± 0.04	0.47
	F143S	0.87 ± 0.04	0.42
	W169S	0.25 ± 0.06	0.12
	R67S/T90A	4.01 ± 0.28	1.94

Values are mean ± SD of three replicates

Among the other mutants, the activity of L47T and Q56T improved to a certain degree, and the mutant G63T had slightly reduced activity. The mutation of F143 to T or S resulted in appreciably impaired activity (Table 2). The residue N141 in CTendo45, homologous to N119 in TtCel45A, was adjacent to the flexible V–VI loop, which is acknowledged as an active regulatory switch in cooperation with the catalytic acid D144 during catalytic reactions (Fig. 1) [28]. Therefore, the addition of a glycan chain at N141 actually blocked the dynamic active space and seriously impeded the specific function of the V–VI loop. Residue W169 is a significant component of the linker joining two α-helices; as a result, the additional branched N-glycan at N167 would destroy the structural configuration [36]. Alternatively, the additional glycan can generate a steric hindrance to perturb the ability of the catalytic base D32 (Fig. 1) [40], resulting in a near-complete loss of the enzyme’s ability to hydrolyze both CMC-Na and β-d-glucan.

As mentioned above, N-glycosylation at different positions exerted diverse effects on enzyme activity, which is consistent with the results of recent studies on other glucoside hydrolases [17, 37, 41]. Residue substitutions at either the F143 or W169 position had a strong negative influence on enzymatic activity (Table 2); hence, the mutants F143S, F143T, and W169S were not further analyzed in this study.

### Effect of N-glycosylation on thermostability

To determine the effect of glycosylation on enzyme thermostability, the hydrolytic activities of these endoglucanases were detected after preincubation at different temperatures ranging from 30 to 90 °C for 200 min. T90A exhibited excellent heat resistance after treatment at high temperatures with CMC-Na (Fig. 4c). Consistent thermostability results were realized for T90A using β-d-glucan as a substrate (Additional file 1: Fig. S5). Among the other single mutants, L47T and R67S were more thermostable than CTendo45 at elevated temperatures, while both Q56T and G63T lost nearly all hydrolytic activity after 200 min of incubation at 90 °C with each substrate. The high-temperature resistance of the double-mutant R67S/T90S was much greater than that of the single-mutation counterparts. Furthermore, the half-lives (*t*_1/2_) consistently revealed that four mutants, L47T, R67S, T90A, and R67S/T90S, exhibited improved enzyme thermostability at 80 °C and 90 °C (Table 3).

**Table 3:** Half-life of CTendo45 and its mutants in thermal inactivation

Substrate	Enzyme	*t*_1/2_ and residual relative activity	
		80 °C	90 °C
CMC-Na	CTendo45	150 min (48.8%)	35 min (52.9%)
	L47T	230 min (53.2%)	55 min (51.3%)
	Q56T	110 min (52.2%)	20 min (46.2%)
	G63T	130 min (48.5%)	25 min (47.5%)
	R67S	190 min (51.7%)	45 min (49.2%)
	T90A	260 min (47.5%)	65 min (52.4%)
	R67S/T90A	320 min (47.8%)	85 min (48.3%)
β-d-glucan	CTendo45	130 min (52.6%)	28 min (49.8%)
	L47T	210 min (53.6%)	45 min (52.3%)
	Q56T	100 min (52.6%)	15 min (54.2%)
	G63T	110 min (53.6%)	20 min (48.6%)
	R67S	160 min (47.1%)	35 min (53.5%)
	T90A	230 min (51.7%)	50 min (49.5%)
	R67S/T90A	270 min (53.0%)	65 min (48.3%)

Untreated enzyme was considered as the control (100%)

Previous studies have demonstrated that the optimized stabilization introduced by additional glycosylation is closely associated with entropy, which is largely dependent on the positions of glycosylation sites [38, 42]. Glycans attached to the flexible region within random coils would, in general, inherently confine the conformational space and encourage entropic reduction to a point to enhance conformational stability at high temperatures [17, 36]. Additionally, the polarity of the protein surface would significantly change after glycosylation, extending the tertiary structure and exposing some hydrophobic amino acids to a more hydrophilic environment [43]. Nevertheless, the lower thermostability of Q56T and G63T could be related to other complicated structural determinants that increase the protein’s entropy, for instance, the destruction of hydrogen bonds and the perturbation of secondary structures through deglycosylation [44, 45]. The greater thermostability of T90A appeared to be connected with the amino acid position located in the backbone structure of the β-barrel region. The conjugated glycan might have acted as a strong clamp and tightened the enzyme spatial conformation with increased configurational entropy [38]. Thus, the reduction in the entropy of the folded state, in the case of deglycosylation, might repress fluctuations in a more stable structure and thereby reduce heat sensitivity [46]. Thermostability is a complex property that can be controlled by several factors; therefore, the thermodynamic mechanism has not yet been fully elucidated [5]. In this case, additional details should be pursued in future research to ascertain the reasons for the enhanced thermostability resulting from the modification of N-glycosylation sites.

### Effect of N-glycosylation on kinetic characterization

The kinetic parameters against CMC-Na were determined at the optimum enzyme conditions of 60 °C and pH 4 (Table 4). The catalytic efficiency of T90A was significantly increased, as the kcat/Km value was 1.57-fold greater than that of native CTendo45, and this trend was consistent for the Vmax and kcat values of T90A. The additional mutant R67S also showed an obvious increase in turnover rate and catalytic efficiency with elevated kcat and kcat/Km values. The double-mutant R67S/T90S inherited the improved enzymatic performance from its single-mutation counterparts. In contrast, the mutant G63T had a reduction in catalytic efficiency, and the kcat/Km value was lower than that of other endoglucanases. The kinetic parameters of each enzyme were also measured with barley β-d-glucan as the native substrate (Table 4). These results indicated that the N-glycosylation modifications, except for that of G63T, effectively improved the catalytic efficiency towards cellulose substrates.

**Table 4:** Kinetic parameters of CTendo45 and its mutants against CMC-Na and β-d-glucan

Substrate	Enzyme	Km (mg/mL)	Vmax (µg/min/mL)	kcat (s^−1^)	kcat/Km (µL/s/mg)
CMC-Na	CTendo45	5.93 ± 0.54	4.42 ± 0.15	0.37 ± 0.04	62.06 ± 1.25
	L47T	3.28 ± 0.12	3.77 ± 0.03	0.25 ± 0.03	74.65 ± 1.36
	Q56T	2.23 ± 0.41	3.89 ± 0.42	0.16 ± 0.03	70.67 ± 1.74
	G63T	20.90 ± 1.27	9.84 ± 0.80	0.75 ± 0.05	35.68 ± 0.97
	R67S	4.75 ± 0.20	8.21 ± 1.03	0.40 ± 0.08	83.49 ± 1.25
	T90A	10.81 ± 0.05	15.87 ± 0.05	1.05 ± 0.12	97.54 ± 2.13
	R67S/T90A	13.73 ± 0.06	18.66 ± 0.09	1.55 ± 0.07	112.9 ± 3.72
β-d-glucan	CTendo45	29.11 ± 3.68	45.95 ± 5.68	4.03 ± 0.51	138.46 ± 2.07
	L47T	6.28 ± 0.94	14.50 ± 1.19	1.21 ± 0.14	192.43 ± 1.85
	Q56T	16.68 ± 1.89	38.68 ± 4.06	2.96 ± 0.18	177.29 ± 1.47
	G63T	56.57 ± 8.82	112.45 ± 16.10	7.94 ± 0.59	140.39 ± 1.64
	R67S	17.03 ± 1.75	44.07 ± 5.21	3.38 ± 0.42	198.76 ± 2.17
	T90A	28.34 ± 2.73	66.58 ± 5.40	6.18 ± 0.75	217.90 ± 2.83
	R67S/T90A	57.73 ± 4.59	67.4 ± 2.87	13.95 ± 2.40	241.63 ± 18.69

Values are mean ± SD of three replicates

The covalent bond connecting the oligosaccharide and the N-glycosylation site could adjust the energy landscape of glycoproteins, resulting in substantial changes in kinetic properties [47]. The deglycosylated mutant exhibited increased catalytic efficiency, probably due to a relatively flexible protein structure that in turn influenced the location and function of amino acids at the active site [48]. The residue N65 is located near the active site cleft, and the branched N-glycan would unavoidably interact with cellulose chains. To tear away a single chain from the surface of cellulose molecules, multiple intermolecular hydrogen bonds must be formed, and the free energy of new hydrogen bonds can facilitate the hydrolytic process [17]. The discrepancies in kinetic characterization among L47T, Q56T, and G63T are mainly attributed to their different amino acid positions (although all of them are situated in the flexible loop), where the glycan would play different roles in enzyme properties [41].

From a practical perspective, the efficient catalytic activity of enzymes is a crucial prerequisite for industrial applications. Consequently, T90A, R67S, and their double-mutant R67S/T90A, which showed considerable thermostability and elevated catalytic efficiency, are regarded as prospective candidates for widespread biotechnological applications. More remarkably, the N88- and N65-glycosylation sites are highly conserved in homologous sequences of CTendo45. Therefore, the design principle regarding the use of N-glycosylation site modification to optimize enzyme properties may be widely applied to homologous endoglucanases sharing a similar structure with CTendo45 or even to the majority of GH45 family members.

## Conclusion

In this study, we investigated for the first time each modifiable N-glycosylation site of a thermostable endoglucanase and obtained three mutants, T90A, R67S, and the double-mutant R67S/T90A, with superior catalytic activity and thermostability compared to the wild-type and other mutants. This work provides preliminary insight into the biological functions of N-glycosylation in thermostable endoglucanases and has referential significance for engineering homologous and structurally similar enzymes to improve enzymatic performance via rational design.

## Methods

### Materials

*Escherichia coli* T1 (TransGen, Beijing, China) was used for gene cloning and sequencing. *Pichia pastoris* GS115 (Invitrogen, Carlsbad, CA, USA) was used as a heterologous expression host. The pPIC9K vector (Invitrogen) was used for constitutive expression in *P. pastoris*. The recombinant plasmid pPIC9K/*ctendo45*, harboring the endoglucanase-encoding gene *ctendo45* (GenBank Accession no. KC441877) and a 6× His-tag at the C-terminus, was previously constructed [12]. A Fast Mutagenesis System Kit was purchased from TransGen. Mutagenic primers were synthesized by Sangon (Shanghai, China) and are summarized in Additional file 1: Table S4. All chemicals were of analytical grade.

### Mutagenesis of CTendo45

N-glycosylation site and O-glycosylation site analyses were carried out using the NetNGlyc 1.0 Server (http://www.cbs.dtu.dk/services/NetNGlyc/) and the NetOGlyc 4.0 Server (http://www.cbs.dtu.dk/services/NetOGlyc/), respectively. Homologous sequence alignment was performed using Clustal Omega (https://www.ebi.ac.uk/Tools/msa/clustalo/). The homology-modeled structure of the *Thielavia terrestris* β-1,4-endoglucanase TtCel45A (PDB: 5GLY) was used to predict the biological function of candidate mutation sites [28]. Homologous modeling was performed using the online software SWISS-MODEL. To remove the existing N-glycosylation sites and introduce additional ones, different residues were selected to produce one deletion mutant (T90A), seven single addition mutants (L47T, Q56T, G63T, R67S, F143T, F143S and W169S), and one double mutant (R67S/T90A). Each target mutant plasmid was generated by site-directed mutagenesis with the plasmid pPIC9K/*ctendo45* as a PCR template and then transformed into *E. coli* T1. Positive colonies were picked on LB agar plates supplemented with 50 µg/mL kanamycin after culture at 37 °C for 14 h and ultimately verified by DNA sequencing with AOX1 gene primers and self-primers (Additional file 1: Table S4).

### Heterologous expression in *Pichia pastoris*

The identified recombinant plasmid was linearized using SacI (Fermentas, Thermo Scientific, Waltham, MA, USA) and electrotransformed into *P. pastoris* GS115 [49]. The transformants that emerged on MD and MM plates were inoculated onto YPD medium plates supplemented with 1–4 mg/mL G418 (Sangon) and cultured at 28 °C for 3 days to select multicopy integrants [12]. PCR amplification was performed with AOX1 primers based on the genomic DNA extracted from the acquired multicopy colony to confirm the presence of the mutant plasmid. Enzyme induction was carried out according to the protocol of a *Pichia* Expression Kit (Invitrogen) [50].

### Purification and SDS-PAGE analysis

After 7 days of methanol-induced culture, the cell-free culture supernatant was collected by centrifugation at 8000 rpm for 15 min. Then, the supernatant was precipitated with ammonium sulfate at 80% saturation and 4 °C overnight. The suspension was centrifuged at 8000 rpm for 15 min and the precipitate was dissolved in 20 mM phosphate buffer solution (pH 7.4). Afterwards, His-tagged recombinant enzymes were purified using Ni^2+^ affinity chromatography (HisTrap™ FF crude; GE Healthcare, Buckinghamshire, UK), as previously described [49]. Protein concentrations were estimated using a Pierce™ BCA Protein Assay Kit (Thermo Scientific). SDS-PAGE analysis was carried out in a 12% (w/v) polyacrylamide gel, and staining was conducted with Coomassie blue R-250 (Sigma-Aldrich, St. Louis, MO, USA) and a Pierce™ Glycoprotein Staining Kit (Thermo Scientific), respectively. PNGase F, which is the most effective enzyme for specifically removing N-linked glycans (but not O-linked glycans) from glycoproteins [51], was obtained from New England Biolabs (Ipswich, MA, USA).

### Enzymatic activity assay

β-d-glucan and CMC-Na (400–800 centipoise in water at room temperature) were purchased from Sigma-Aldrich as substrates. The reaction system comprised 150 µL of 1% (w/v) CMC-Na or 0.2% (w/v) β-d-glucan and 15 µg of purified enzyme in a 300 µL reaction mixture. The reaction was incubated at 60 °C for 30 min and terminated by the addition of 300 µL of 3,5-dinitrosalicylic acid reagent in a boiling water bath for exactly 10 min. After the sample was cooled to ambient temperature, the absorbance was read at 540 nm [52]. One international unit (IU) of enzyme activity was defined as the amount of enzyme that catalyzes the liberation of reducing sugar equivalent to 1 μmoL of glucose per minute under the reaction conditions. Each experiment was performed in triplicate.

### Biochemical characterization

The optimal pH was measured in multiple buffer solutions at 50 mM concentrations, including acetate buffer (pH 3–6), sodium phosphate buffer (pH 6–8), and Tris–HCl buffer (pH 8–9). The optimal temperature was evaluated from 30 to 90 °C at the optimal pH value [53]. Thermostability was determined by detecting the residual enzyme activity after the enzyme was preincubated at 30–90 °C for 200 min [54]. Moreover, the half-life (*t*_1/2_), which was defined as the time at which the enzyme activity declined to half of its initial activity value at that temperature, was investigated at 80 °C and 90 °C [55].

### Kinetic parameters

The reaction was performed in 50 mM acetate buffer (pH 4) at 60 °C for 30 min using an appropriate equivalent amount of diluted enzyme with varying concentrations of CMC-Na (1–10 mg/mL) and β-d-glucan (0.5–5 mg/mL). Kinetic parameters were calculated according to the Michaelis–Menten equation [56].

## Supplementary information

**Additional file 1: Table S1.** Glycosylation sites analysis of CTendo45 and mutants using the NetNGlyc 1.0 Server. **Table S2.** Protein yields of CTendo45 and its mutants after purification. **Table S3.** N-glycosylated peptides and glycan structures found in CTendo45 and its mutants. **Table S4.** Nucleotide sequences of primers used in this study. **Fig. S1.** DNA sequences of CTendo45 and designed mutants. **Fig. S2.** Translated amino acids sequences of CTendo45 and designed mutants. **Fig. S3.** SDS-PAGE analysis of purified recombinant enzymes. **Fig. S4.** Mass spectrum of tryptic digest of CTendo45 and its mutants. **Fig. S5.** Enzymatic properties of wild-type CTendo45 and its mutants against β-d-glucan. **Fig. S6.** Characterization of non-glycosylated versions of CTendo45.

## Data Availability

The data sets supporting the conclusions of this article are included within the article (and its Additional file 1).

## Abbreviations

PNGase F: Peptide-N-glycosidase
CMC-Na: Carboxymethylcellulose sodium
SDS-PAGE: Sodium dodecyl sulfate-polyacrylamide gel electrophoresis
LB: Luria-Bertani
MD: Minimal dextrose
MM: Minimal methanol
YPD: Yeast peptone dextrose

## References

[1] Kubicek CP, Kubicek EM (2016). Enzymatic deconstruction of plant biomass by fungal enzymes. Curr Opin Chem Biol.

[2] Payne CM, Knott BC, Mayes HB, Hansson H, Himmel ME, Sandgren M, Ståhlberg J, Beckham GT (2015). Fungal cellulases. Chem Rev.

[3] Wang X, Zeng J, Gao W, Chen K, Wang B, Xu J (2019). Endoglucanase recycling for disintegrating cellulosic fibers to fibrils. Carbohydr Polym.

[4] Yennamalli RM, Rader AJ, Kenny AJ, Wolt JD, Sen TZ (2013). Endoglucanases: insights into thermostability for biofuel applications. Biotechnol Biofuels.

[5] Patel AK, Singhania RR, Sim SJ, Pandey A (2019). Thermostable cellulases: current status and perspectives. Bioresour Technol.

[6] Deshpande S, Masurkar ND, Girish VM, Desai M, Chakraborty G, Chan JM, Drum CL (2017). Thermostable exoshells fold and stabilize recombinant proteins. Nat Commun.

[7] Liu CG, Xiao Y, Xia XX, Zhao XQ, Peng L, Srinophakun P, Bai FW (2019). Cellulosic ethanol production: progress, challenges and strategies for solutions. Biotechnol Adv.

[8] Srivastava N, Srivastava M, Mishra PK, Gupta VK, Molina G, Rodriguez-Couto S, Ambepu Manikanta A, Ramteke PW (2018). Applications of fungal cellulases in biofuel production: advances and limitations. Renew Sustain Energy Rev.

[9] Liu Q, Xun G, Feng Y (2019). The state-of-the-art strategies of protein engineering for enzyme stabilization. Biotechnol Adv.

[10] Taylor LE, Knott BC, Baker JO, Alahuhta PM, Hobdey SE, Linger JG, Lunin VV, Amore A, Subramanian V, Podkaminer K, Xu Q, VanderWall TA, Schuster LA, Chaudhari YB, Adney WS, Crowley MF, Himmel ME, Decker SR, Beckham GT (2018). Engineering enhanced cellobiohydrolase activity. Nat Commun.

[11] Bashirova A, Pramanik S, Volkov P, Rozhkova A, Nemashkalov V, Zorov I, Gusakov A, Sinitsyn A, Schwaneberg U, Davari MD (2019). Disulfide bond engineering of an endoglucanase from *Penicillium verruculosum* to improve its thermostability. Int J Mol Sci.

[12] Chen X, Li W, Ji P, Zhao Y, Hua C, Han C (2018). Engineering the conserved and noncatalytic residues of a thermostable β-1,4-endoglucanase to improve specific activity and thermostability. Sci Rep.

[13] Torktaz I, Karkhane AA, Hemmat J (2018). Rational engineering of Cel5E from *Clostridium thermocellum* to improve its thermal stability and catalytic activity. Appl Microbiol Biotechnol.

[14] Zheng F, Tu T, Wang X, Wang Y, Ma R, Su X, Xie X, Yao B, Luo H (2018). Enhancing the catalytic activity of a novel GH5 cellulase *Gt*Cel5 from *Gloeophyllum trabeum* CBS 900.73 by site-directed mutagenesis on loop 6. Biotechnol Biofuels.

[15] Wild R, Kowal J, Eyring J, Ngwa EM, Aebi M, Locher KP (2018). Structure of the yeast oligosaccharyltransferase complex gives insight into eukaryotic N-glycosylation. Science.

[16] Amore A, Knott BC, Supekar NT, Shajahan A, Azadi P, Zhao P, Wells L, Linger JG, Hobdey SE, Vander Wall TA, Shollenberger T, Yarbrough JM, Tan Z, Crowley MF, Himmel ME, Decker SR, Beckham GT, Taylor LE (2017). Distinct roles of N- and O-glycans in cellulase activity and stability. Proc Natl Acad Sci USA.

[17] Dotsenko AS, Gusakov AV, Volkov PV, Rozhkova AM, Sinitsyn AP (2016). N-linked glycosylation of recombinant cellobiohydrolase I (Cel7A) from *Penicillium verruculosum* and its effect on the enzyme activity. Biotechnol Bioeng.

[18] Gao L, Gao F, Wang L, Geng C, Chi L, Zhao J, Qu Y (2012). N-glycoform diversity of cellobiohydrolase I from *Penicillium decumbens* and synergism of nonhydrolytic glycoform in cellulose degradation. J Biol Chem.

[19] Gusakov AV, Dotsenko AS, Rozhkova AM, Sinitsyn AP (2017). N-Linked glycans are an important component of the processive machinery of cellobiohydrolases. Biochimie.

[20] Qi F, Zhang W, Zhang F, Chen G, Liu W (2014). Deciphering the effect of the different N-glycosylation sites on the secretion, activity, and stability of cellobiohydrolase I from *Trichoderma reesei*. Appl Environ Microbiol.

[21] Kar B, Verma P, den Haan R, Sharma AK (2018). Effect of N-linked glycosylation on the activity and stability of a β-glucosidase from *Putranjiva roxburghii*. Int J Biol Macromol.

[22] Wei W, Chen L, Zou G, Wang Q, Yan X, Zhang J, Wang C, Zhou Z (2013). N-glycosylation affects the proper folding, enzymatic characteristics and production of a fungal β-glucosidase. Biotechnol Bioeng.

[23] Li W, Ji P, Zhou Q, Hua C, Han C (2018). Insights into the synergistic biodegradation of waste papers using a combination of thermostable endoglucanase and cellobiohydrolase from *Chaetomium thermophilum*. Mol Biotechnol.

[24] Zhou Q, Ji P, Zhang J, Li X, Han C (2017). Characterization of a novel thermostable GH45 endoglucanase from *Chaetomium thermophilum* and its biodegradation of pectin. J Biosci Bioeng.

[25] Haddad Y, Heger Z, Adam V (2017). Targeting neuroblastoma cell surface proteins: recommendations for homology modeling of hNET, ALK, and TrkB. Front Mol Neurosci.

[26] Isoherranen N, Zhong G (2019). Biochemical and physiological importance of the CYP26 retinoic acid hydroxylases. Pharmacol Ther.

[27] Davies GJ, Dodson GG, Hubbard RE, Tolley SP, Dauter Z, Wilson KS, Hjort C, Mikkelsen JM, Rasmussen G, Schülein M (1993). Structure and function of endoglucanase V. Nature.

[28] Gao J, Huang JW, Li Q, Liu W, Ko TP, Zheng Y, Xiao X, Kuo CJ, Chen CC, Guo RT (2017). Characterization and crystal structure of a thermostable glycoside hydrolase family 45 1,4-β-endoglucanase from *Thielavia terrestris*. Enzyme Microb Technol.

[29] Hirvonen M, Papageorgiou AC (2003). Crystal structure of a family 45 endoglucanase from *Melanocarpus albomyces*: mechanistic implications based on the free and cellobiose-bound forms. J Mol Biol.

[30] Nakamura A, Ishida T, Kusaka K, Yamada T, Fushinobu S, Tanaka I, Kaneko S, Ohta K, Tanaka H, Inaka K, Higuchi Y, Niimura N, Samejima M, Igarashi K (2015). “Newton’s cradle” proton relay with amide-imidic acid tautomerization in inverting cellulase visualized by neutron crystallography. Sci Adv.

[31] Sargsyan K, Grauffel C, Lim C (2017). How molecular size impacts RMSD applications in molecular dynamics simulations. J Chem Theory Comput.

[32] Waterhouse A, Bertoni M, Bienert S, Studer G, Tauriello G, Gumienny R, Heer FT, de Beer TAP, Rempfer C, Bordoli L, Lepore R, Schwede T (2018). SWISS-MODEL: homology modelling of protein structures and complexes. Nucleic Acids Res.

[33] Cha JH, Yoon JJ, Cha CJ (2018). Functional characterization of a thermostable endoglucanase belonging to glycoside hydrolase family 45 from *Fomitopsis palustris*. Appl Microbiol Biotechnol.

[34] Davies GJ, Tolley SP, Henrissat B, Hjort C, Schulein M (1995). Structures of oligosaccharide-bound forms of the endoglucanase V from *Humicola insolensat* 1.9 Å resolution. Biochemistry.

[35] Bause E (1983). Structural requirements of N-glycosylation of proteins. Studies with proline peptides as conformational probes. Biochem J.

[36] Adney WS, Jeoh T, Beckham GT, Chou YC, Baker JO, Michener W, Brunecky R, Himmel ME (2009). Probing the role of N-linked glycans in the stability and activity of fungal cellobiohydrolases by mutational analysis. Cellulose.

[37] Mak WS, Siegel JB (2014). Computational enzyme design: transitioning from catalytic proteins to enzymes. Curr Opin Struct Biol.

[38] Shental-Bechor D, Levy Y (2008). Effect of glycosylation on protein folding: a close look at thermodynamic stabilization. Proc Natl Acad Sci USA.

[39] Chen L, Drake MR, Resch MG, Greene ER, Himmel ME, Chaffey PK, Beckham GT, Tan Z (2014). Specificity of *O*-glycosylation in enhancing the stability and cellulose binding affinity of family 1 carbohydrate-binding modules. Proc Natl Acad Sci USA.

[40] Valjakka J, Rouvinen J (2003). Structure of 20 K endoglucanase from *Melanocarpus albomyces* at 1.8 Å resolution. Acta Crystallogr D Biol Crystallogr.

[41] Fonseca-Maldonado R, Vieira DS, Alponti JS, Bonneil E, Thibault P, Ward RJ (2013). Engineering the pattern of protein glycosylation modulates the thermostability of a GH11 xylanase. J Biol Chem.

[42] Helenius A, Aebi M (2004). Roles of N-linked glycans in the endoplasmic reticulum. Annu Rev Biochem.

[43] Wang Z, Guo C, Liu L, Huang H (2018). Effects of N-glycosylation on the biochemical properties of recombinant bEKL expressed in Pichia pastoris. Enzyme Microb Technol.

[44] Huang JW, Cheng YS, Ko TP, Lin CY, Lai HL, Chen CC, Ma Y, Zheng Y, Huang CH, Zou P, Liu JR, Guo RT (2012). Rational design to improve thermostability and specific activity of the truncated *Fibrobacter succinogenes* 1,3-1,4-β-d-glucanase. Appl Microbiol Biotechnol.

[45] Sriprapundh D, Vieille C, Zeikus JG (2000). Molecular determinants of xylose isomerase thermal stability and activity: analysis of thermozymes by site-directed mutagenesis. Protein Eng.

[46] Matthews BW, Nicholson H, Becktel WJ (1987). Enhanced protein thermostability from site-directed mutations that decrease the entropy of unfolding. Proc Natl Acad Sci USA.

[47] Moser JW, Wilson IBH, Dragosits M (2017). The adaptive landscape of wild-type and glycosylation-deficient populations of the industrial yeast *Pichia pastoris*. BMC Genomics.

[48] Live DH, Kumar RA, Beebe X, Danishefsky SJ (1996). Conformational influences of glycosylation of a peptide: a possible model for the effect of glycosylation on the rate of protein folding. Proc Natl Acad Sci USA.

[49] Hua C, Li W, Han W, Wang Q, Bi P, Han C, Zhu L (2018). Characterization of a novel thermostable GH7 endoglucanase from *Chaetomium thermophilum* capable of xylan hydrolysis. Int J Biol Macromol.

[50] Cereghino JL, Cregg JM (2000). Heterologous protein expression in the methylotrophic yeast *Pichia pastoris*. FEMS Microbiol Rev.

[51] Huang C, Harada Y, Hosomi A, Masahara-Negishi Y, Seino J, Fujihira H, Funakoshi Y, Suzuki T, Dohmae N, Suzuki T (2015). Endo-β-*N*-acetylglucosaminidase forms N-GlcNAc protein aggregates during ER-associated degradation in Ngly1-defective cells. Proc Natl Acad Sci USA.

[52] Miller GL (1959). Use of dinitrosalicylic acid reagent for determination of reducing sugar. Anal Chem.

[53] Ribeiro LF, De Lucas RC, Vitcosque GL, Ribeiro LF, Ward RJ, Rubio MV, Damásio AR, Squina FM, Gregory RC, Walton PH, Jorge JA, Prade RA, Buckeridge MS, Polizeli Mde L (2014). A novel thermostable xylanase GH10 from *Malbranchea pulchella* expressed in *Aspergillus nidulans* with potential applications in biotechnology. Biotechnol Biofuels.

[54] Phadtare P, Joshi S, Satyanarayana T (2017). Recombinant thermo-alkali-stable endoglucanase of *Myceliopthora thermophila* BJA (rMt-egl): biochemical characteristics and applicability in enzymatic saccharification of agro-residues. Int J Biol Macromol.

[55] Liu X, Liang M, Liu Y, Fan X (2017). Directed evolution and secretory expression of a pyrethroid-hydrolyzing esterase with enhanced catalytic activity and thermostability. Microb Cell Fact.

[56] Carlin DA, Caster RW, Wang X, Betzenderfer SA, Chen CX, Duong VM, Ryklansky CV, Alpekin A, Beaumont N, Kapoor H, Kim N, Mohabbot H, Pang B, Teel R, Whithaus L, Tagkopoulos I, Siegel JB (2016). Kinetic characterization of 100 glycoside hydrolase mutants enables the discovery of structural features correlated with kinetic constants. PLoS ONE.

