# Supplementary material for: Improvement of the catalytic activity and thermostability of a hyperthermostable endoglucanase by optimizing N-glycosylation sites
Source: Biotechnol Biofuels. 2020 Feb 26;13:30. doi: 10.1186/s13068-020-1668-4 (PMC7045587; doi:10.1186/s13068-020-1668-4)
Supplement: Supplementary file 1 — Additional file 1: Table S1. Glycosylation sites analysis of CTendo45 and mutants using the NetNGlyc 1.0 Server. Table S2. Protein yields of CTendo45 and its mutants after purification. Table S3. N-glycosylated peptides and glycan structures found in CTendo45 and its mutants. Table S4. Nucleotide sequences of primers used in this study. Fig. S1. DNA sequences of CTendo45 and designed mutants. Fig. S2. Translated amino acids sequences of CTendo45 and designed mutants. Fig. S3. SDS-PAGE analysis of purified recombinant enzymes. Fig. S4. Mass spectrum of tryptic digest of CTendo45 and its mutants. Fig. S5. Enzymatic properties of wild-type CTendo45 and its mutants against β-d-glucan. Fig. S6. Characterization of non-glycosylated versions of CTendo45. [file 13068_2020_1668_MOESM1_ESM.docx]

**Additional file**

**Improvement of the catalytic activity and thermostability of a hyperthermostable endoglucanase by optimizing N-glycosylation sites**

Chao Han*, Qunqing Wang, Yanxu Sun, Ruirui Yang, Mengyu Liu, Siqi Wang, Yifan Liu, Lifan Zhou and Duochuan Li*

*Correspondence: [hanch87@163.com](mailto:hanch87@163.com); lidc20@sdau.edu.cn

Shandong Key Laboratory for Agricultural Microbiology, College of Plant Protection, Shandong Agricultural University, Tai’an, Shandong 271018, China

Additional file 1 includes:

Tables S1-S4

Figures S1-S6

**Table S1 N-glycosylation sites analysis of CTendo45 and** **mutants using the NetNGlyc 1.0 Server**

| **Protein** | **Position** | **Potential** | **Jury agreement** | **N-Glyc result** |
| --- | --- | --- | --- | --- |
| CTendo45 | 81 NQSP | 0.1806 | (9/9) | --- |
|  | 88 NETL | 0.7536 | (9/9) | +++ |
| L47T | 45 NATQ | 0.7487 | (9/9) | ++ |
|  | 81 NQSP | 0.1806 | (9/9) | --- |
|  | 88 NETL | 0.7536 | (9/9) | +++ |
| Q56T | 54 NATD | 0.6636 | (9/9) | ++ |
|  | 81 NQSP | 0.1806 | (9/9) | --- |
|  | 88 NETL | 0.7536 | (9/9) | +++ |
| G63T | 61 NDTG | 0.6406 | (9/9) | ++ |
|  | 81 NQSP | 0.1806 | (9/9) | --- |
|  | 88 NETL | 0.7536 | (9/9) | +++ |
| R67S | 65 NTSS | 0.6127 | (7/9) | + |
|  | 81 NQSP | 0.1806 | (9/9) | --- |
|  | 88 NETL | 0.7536 | (9/9) | +++ |
| T90A | --- | --- | --- | --- |
| F143T | 81 NQSP | 0.1806 | (9/9) | --- |
|  | 88 NETL | 0.7536 | (9/9) | +++ |
|  | 141 NHTD | 0.6663 | (9/9) | ++ |
| F143S | 81 NQSP | 0.1806 | (9/9) | --- |
|  | 88 NETL | 0.7536 | (9/9) | +++ |
|  | 141 NHSD | 0.6069 | (6/9) | + |
| W169S | 81 NQSP | 0.1806 | (9/9) | --- |
|  | 88 NETL | 0.7536 | (9/9) | +++ |
|  | 167 NGSG | 0.5457 | (6/9) | + |
| R67S/T90A | 65 NTSS | 0.6128 | (7/9) | + |
|  | 81 NQSP | 0.1807 | (9/9) | --- |

**Table S2 Protein yields of CTendo45 and its mutants after purification**

| **Enzyme** | **Protein yield (mg/L)** |
| --- | --- |
| CTendo45 | 358.9 ± 46.1 |
| L47T | 324.7 ± 23.4 |
| Q56T | 411.9 ± 31.8 |
| G63T | 352.3 ± 45.2 |
| R67S | 360.8 ± 36.5 |
| T90A | 435.4 ± 37.9 |
| F143T | 385.4 ± 22.7 |
| F143S | 331.0 ± 31.6 |
| W169S | 284.7 ± 22.6 |
| R67S/ T90A | 402.6 ± 15.4 |

Values are means ±SD of three replicates.

**Table S3 N-glycosylated peptides and glycan structures found in CTendo45 and its mutants. These recombinant endoglucanases were digested with trypsin (Sigma-Aldrich, St. Louis, MO, USA) and the resulting peptides were detected by UHPLC-LTQ-Orbitrap-MS/MS (Thermo Fisher Scientific, Waltham, MA, USA). The data was analyzed by using the GlycoMod tool (**[**http://web.expasy.org/glycomod/**](http://web.expasy.org/glycomod/)**)**

| **Enzyme** | **Glycosylation site** | **Peptide** | **Glycan structure** | **Glycopeptide mass^a^** | **Observed m/z^b^** | **z value^c^** |
| --- | --- | --- | --- | --- | --- | --- |
| CTendo45 | Asn88 | Ser68-Arg99 | (Hex)_1_ (HexNAc)_1_ + (Man)_3_(GlcNAc)_2_ | 4609.9 | 354.6 | 13 |
|  |  |  | (Hex)_1_ (HexNAc)_10_ | 5561.3 | 370.9 | 15 |
|  |  |  | (HexNAc)_8_ + (Man)_3_(GlcNAc)_2_ | 5869.4 | 391.3 | 15 |
|  |  |  | (Hex)_3_ (HexNAc)_16_ + (Man)_3_(GlcNAc)_2_ | 8012.2 | 445.1 | 18 |
|  |  |  | (Hex)_2_ (HexNAc)_3_ | 4285.8 | 475.8 | 9 |
|  |  |  | (Hex)_11_ (HexNAc)_6_ + (Man)_3_(GlcNAc)_2_ | 7261.8 | 519.1 | 14 |
|  |  |  | (Hex)_8_ (HexNAc)_12_ + (Man)_3_(GlcNAc)_2_ | 8010.1 | 571.8 | 14 |
|  |  |  | (Hex)_5_ (HexNAc)_11_ + (Man)_3_(GlcNAc)_2_ | 7320.9 | 610.2 | 12 |
|  |  |  | (Hex)_1_ (HexNAc)_9_ + (Man)_3_(GlcNAc)_2_ | 6242.5 | 624.2 | 10 |
|  |  |  | (Hex)_7_ (HexNAc)_6_ + (Man)_3_(GlcNAc)_2_ | 6588.1 | 658.8 | 10 |
|  |  |  | (Hex)_9_ | 4826.9 | 803.5 | 6 |
|  |  |  | (Hex)_15_ (HexNAc)_8_ + (Man)_3_(GlcNAc)_2_ | 8332.2 | 833.2 | 10 |
| L47T | Asn45 | Ser44-Arg67 | (Hex)_3_ (HexNAc)_1_ | 3218.4 | 536.2 | 6 |
|  |  |  | (Hex)_2_ (HexNAc)_6_ + (Man)_3_(GlcNAc)_2_ | 4964.0 | 551.5 | 9 |
|  |  |  | (Hex)_14_(HexNAc)_2_ + (Man)_3_(GlcNAc)_2_ | 6069.4 | 1014.6 | 6 |
|  |  |  | (Hex)_2_ (HexNAc)_3_ + (Man)_3_(GlcNAc)_2_ | 4354.8 | 1087.4 | 4 |
|  | Asn88 | Ser68-Arg99 | (Hex)_1_ (HexNAc)_1_+ (Man)_3_(GlcNAc)_2_ | 4609.9 | 354.6 | 13 |
|  |  |  | (Hex)_1_ (HexNAc)_10_ | 5561.3 | 370.9 | 15 |
|  |  |  | (HexNAc)_8_ + (Man)_3_(GlcNAc)_2_ | 5869.4 | 391.3 | 15 |
|  |  |  | (Hex)_3_ (HexNAc)_16_ + (Man)_3_(GlcNAc)_2_ | 8012.2 | 445.1 | 18 |
|  |  |  | (Hex)_1_(HexNAc)_3_ | 4285.8 | 475.8 | 9 |
|  |  |  | (Hex)_2_ (HexNAc)_10_ | 5723.3 | 476.8 | 12 |
|  |  |  | (Hex)_11_ (HexNAc)_6_ + (Man)_3_(GlcNAc)_2_ | 7261.8 | 519.1 | 14 |
|  |  |  | (Hex)_8_ (HexNAc)_12_ + (Man)_3_(GlcNAc)_2_ | 8010.1 | 571.8 | 14 |
|  |  |  | (Hex)_5_(HexNAc)_11_ + (Man)_3_(GlcNAc)_2_ | 7320.9 | 610.2 | 12 |
|  |  |  | (Hex)_2_ + (Man)_3_(GlcNAc)_2_ | 4584.9 | 764.7 | 6 |
|  |  |  | (Hex)_1_ (HexNAc)_4_ | 4326.8 | 864.7 | 5 |
| Q56T | Asn54 | Ser44-Arg67 | (Hex)_1_ (HexNAc)_1_ | 2879.3 | 410.8 | 7 |
|  |  |  | (Hex)_3_ (HexNAc)_2_ | 3406.5 | 425.8 | 8 |
|  |  |  | (Hex)_3_(HexNAc)_1_ | 3218.4 | 536.2 | 6 |
|  |  |  | (Hex)_14_(HexNAc)_2_+ (Man)_3_(GlcNAc)_2_ | 6069.4 | 1014.6 | 6 |
|  | Asn88 | Ser68-Arg99 | (Hex)_1_ (HexNAc)_1_+ (Man)_3_(GlcNAc)_2_ | 4609.9 | 354.6 | 13 |
|  |  |  | (Hex)_1_ (HexNAc)_10_ | 5561.3 | 370.9 | 15 |
|  |  |  | (HexNAc)_8_ + (Man)_3_(GlcNAc)_2_ | 5869.4 | 391.3 | 15 |
|  |  |  | (Hex)_3_ (HexNAc)_16_+ (Man)_3_(GlcNAc)_2_ | 8012.2 | 445.1 | 18 |
|  |  |  | (Hex)_2_ (HexNAc)_3_ | 4285.8 | 475.8 | 9 |
|  |  |  | (Hex)_11_ (HexNAc)_6_ + (Man)_3_(GlcNAc)_2_ | 7261.8 | 519.1 | 14 |
|  |  |  | (Hex)_8_ (HexNAc)_12_ + (Man)_3_(GlcNAc)_2_ | 8010.1 | 571.8 | 14 |
|  |  |  | (Hex)_5_ (HexNAc)_11_ + (Man)_3_(GlcNAc)_2_ | 7320.9 | 610.2 | 12 |
|  |  |  | (Hex)_5_ (HexNAc)_2_ + (Man)_3_(GlcNAc)_2_ | 5477.2 | 686.7 | 8 |
|  |  |  | (Hex)_14_(HexNAc)_7_ + (Man)_3_(GlcNAc)_2_ | 7967.0 | 796.7 | 10 |
|  |  |  | (Hex)_11_ (HexNAc)_7_+ (Man)_3_(GlcNAc)_2_ | 7464.9 | 934.7 | 8 |
|  |  |  |  |  |  |  |
| R67S | Asn65 | Ser44-Arg99 | (Hex)_1_ (HexNAc)_14_+ (Man)_3_(GlcNAc)_2_ | 9719.0 | 694.8 | 14 |
|  |  |  | (Hex)_18_ | 8726.5 | 872.7 | 10 |
|  |  |  | (Hex)_11_ (HexNAc)_2_ + (Man)_3_(GlcNAc)_2_ | 8888.6 | 1480.9 | 6 |
|  | Asn88 | Ser44-Arg99 | (Hex)_3_ (HexNAc)_8_ + (Man)_3_(GlcNAc)_2_ | 8778.6 | 548.8 | 16 |
|  |  |  | (Hex)_10_ | 7442.1 | 571.8 | 13 |
|  |  |  | (HexNAc)_3_ + (Man)_3_(GlcNAc)_2_ | 7307.1 | 610.2 | 12 |
|  |  |  | (Hex)_5_ (HexNAc)_16_ + (Man)_3_(GlcNAc)_2_ | 10773.4 | 766.7 | 14 |
|  |  |  | (Hex)_5_ (HexNAc)_1_ + (Man)_3_(GlcNAc)_2_ | 7727.2 | 856.7 | 9 |
| T90A | Asn88^d^ | Ser44-Arg99 | -- | 3321.4 | 553.8 | 6 |
|  |  |  |  | 3321.4 | 664.3 | 5 |
|  |  |  |  | 3321.4 | 830.4 | 4 |
| R67S/T90A | Asn65 | Ser44-Arg99 | (Hex)_1_ (HexNAc)_14_+(Man)_3_(GlcNAc)_2_ | 9719.0 | 694.8 | 14 |
|  |  |  | (Hex)_18_ | 8726.5 | 872.7 | 10 |
|  |  |  | (Hex)_11_(HexNAc)_2_ + (Man)_3_(GlcNAc)_2_ | 8888.6 | 1480.9 | 6 |
|  | Asn88^d^ | Ser44-Arg99 | -- | 3321.4 | 553.8 | 6 |
|  |  |  |  | 3321.4 | 664.3 | 5 |
|  |  |  |  | 3321.4 | 830.4 | 4 |

^a^ The theoretical glycopeptide mass; ^b^ The observed m/z values were remarked in Fig. S4; ^c^ Z stands for charge number; ^d^ The peptide fragments containing unglycosylated N88-E89-A90 motif in T90A and R67S/T90A mutants were detected after mass spectrometry analysis.

**Table S4 Nucleotide sequences of primers used in this study**

| **Primers** | **Sequences (from 5’ to 3’)** |
| --- | --- |
| L47T-F | CGGAAAGTCCAACGCCACGCAACCAGTG |
| L47T-R | GTGGCGTTGGACTTTCCGGGCCAGGCG |
| Q56T-F | GCAAACTTGCAATGCGACGGACCAGCCCCT |
| Q56T-R | GTCGCATTGCAAGTTTGCACTGGTTGC |
| G63T-F | CCAGCCCCTGAACGATACGGGCAACACG |
| G63T-R | GTATCGTTCAGGGGCTGGTCCTGCGCA |
| R67S-F | GATGGGGGCAACACGAGCTCCGGCTG |
| R67S -R | TCGTGTTGCCCCCATCGTTCAGGGGC |
| T90A-F | TGGGCGCTGAACGAGGCACTCTCGTAC |
| T90A-R | CCTCGTTCAGCGCCCAGGGCGATTGG |
| F143T-F | TGGGGAGTAATCATACTGATATTGCT |
| F143T-R | GTATGATTACTCCCCAAATCCCCGCCCG |
| F143S-F | TGGGGAGTAATCATTCTGATATTGCT |
| F143S-R | GAATGATTACTCCCCAAATCCCCGCCCG |
| W169S-F | CCCCTCCAAACGGCTCGGGTGAGCGGT |
| W169S-R | GAGCCGTTTGGAGGGGCGCCGTATTG |
| 5’AOX1 | GACTGGTTCCAATTGACAAGC |
| 3’AOX1 | GCAAATGGCATTCTGACATCC |
| 5’Self-primer | CAAGGTGCCCAAGGCA |
| 3’Self-primer | TTAGGGAGTCCAAGTCG |

**Fig. S1.** DNA sequences of CTendo45 and designed mutants

CTendo45 ATGCATCTCTCTCAGCTTGCCCTCCCCTTGCTCCTCGCTGCGGGTGCTCA 50

L47T -------------------------------------------------- 50

Q56T -------------------------------------------------- 50

G63T -------------------------------------------------- 50

R67S -------------------------------------------------- 50

T90A -------------------------------------------------- 50

F143T -------------------------------------------------- 50

F143S -------------------------------------------------- 50

W169S -------------------------------------------------- 50

R67S/T90A -------------------------------------------------- 50

CTendo45 CGCCCAAGGTGCCCAAGGCACCGGCAGAACAACCCGCTACTGGGATTGCT 100

L47T -------------------------------------------------- 100

Q56T -------------------------------------------------- 100

G63T -------------------------------------------------- 100

R67S -------------------------------------------------- 100

T90A -------------------------------------------------- 100

F143T -------------------------------------------------- 100

F143S -------------------------------------------------- 100

W169S -------------------------------------------------- 100

R67S/T90A -------------------------------------------------- 100

CTendo45 GTAAGCCCTCATGCGCCTGGCCCGGAAAGTCCAACGCCCTGCAACCAGTG 150

L47T --------------------------------------ac---------- 150

Q56T -------------------------------------------------- 150

G63T -------------------------------------------------- 150

R67S -------------------------------------------------- 150

T90A -------------------------------------------------- 150

F143T -------------------------------------------------- 150

F143S -------------------------------------------------- 150

W169S -------------------------------------------------- 150

R67S/T90A -------------------------------------------------- 150

CTendo45 CAAACTTGCAATGCGCAGGACCAGCCCCTGAACGATGGGGGCAACACGCG 200

L47T -------------------------------------------------- 200

Q56T ---------------ac--------------------------------- 200

G63T ------------------------------------ac------------ 200

R67S ------------------------------------------------a- 200

T90A -------------------------------------------------- 200

F143T -------------------------------------------------- 200

F143S -------------------------------------------------- 200

W169S -------------------------------------------------- 200

R67S/T90A ------------------------------------------------a- 200

CTendo45 CTCCGGCTGCGACTCGGGCGGCAGCGCTTTCATGTGCTCAAACCAATCGC 250

L47T -------------------------------------------------- 250

Q56T -------------------------------------------------- 250

G63T -------------------------------------------------- 250

R67S -------------------------------------------------- 250

T90A -------------------------------------------------- 250

F143T -------------------------------------------------- 250

F143S -------------------------------------------------- 250

W169S -------------------------------------------------- 250

R67S/T90A -------------------------------------------------- 250

CTendo45 CCTGGGCGCTGAACGAGACACTCTCGTACGGCTGGGCGGCGGTTAGGATC 300

L47T -------------------------------------------------- 300

Q56T -------------------------------------------------- 300

G63T -------------------------------------------------- 300

R67S -------------------------------------------------- 300

T90A -----------------g-------------------------------- 300

F143T -------------------------------------------------- 300

F143S -------------------------------------------------- 300

W169S -------------------------------------------------- 300

R67S/T90A -----------------g-------------------------------- 300

CTendo45 GCGGGCCAGAGTGAATTCAACTGGTGCTGTGCGTGTTATGAATTGACTTT 350

L47T -------------------------------------------------- 350

Q56T -------------------------------------------------- 350

G63T -------------------------------------------------- 350

R67S -------------------------------------------------- 350

T90A -------------------------------------------------- 350

F143T -------------------------------------------------- 350

F143S -------------------------------------------------- 350

W169S -------------------------------------------------- 350

R67S/T90A -------------------------------------------------- 350

CTendo45 TACCAGTGGGCCGGTGGCGGGGAAGAAGATGATTGTGCAAGCGACGAATA 400

L47T -------------------------------------------------- 400

Q56T -------------------------------------------------- 400

G63T -------------------------------------------------- 400

R67S -------------------------------------------------- 400

T90A -------------------------------------------------- 400

F143T -------------------------------------------------- 400

F143S -------------------------------------------------- 400

W169S -------------------------------------------------- 400

R67S/T90A -------------------------------------------------- 400

CTendo45 CGGGCGGGGATTTGGGGAGTAATCATTTTGATATTGCTATCCCTGGTGGT 450

L47T -------------------------------------------------- 450

Q56T -------------------------------------------------- 450

G63T -------------------------------------------------- 450

R67S -------------------------------------------------- 450

T90A -------------------------------------------------- 450

F143T --------------------------ac---------------------- 450

F143S --------------------------tc---------------------- 450

W169S -------------------------------------------------- 450

R67S/T90A -------------------------------------------------- 450

CTendo45 GGTGTTGGTATCTTCAATGCCTGCACCCAACAATACGGCGCCCCTCCAAA 500

L47T -------------------------------------------------- 500

Q56T -------------------------------------------------- 500

G63T -------------------------------------------------- 500

R67S -------------------------------------------------- 500

T90A -------------------------------------------------- 500

F143T -------------------------------------------------- 500

F143S -------------------------------------------------- 500

W169S -------------------------------------------------- 500

R67S/T90A -------------------------------------------------- 500

CTendo45 CGGCTGGGGTGAGCGGTACGGCGGGATCCGCTCGCGCAGCGAGTGCGACA 550

L47T -------------------------------------------------- 550

Q56T -------------------------------------------------- 550

G63T -------------------------------------------------- 550

R67S -------------------------------------------------- 550

T90A -------------------------------------------------- 550

F143T -------------------------------------------------- 550

F143S -------------------------------------------------- 550

W169S ----c--------------------------------------------- 550

R67S/T90A -------------------------------------------------- 550

CTendo45 GCTTCCCCGAGGCGCTCAAAGCCGGCTGCTACTGGCGTTTCGACTGGTTC 600

L47T -------------------------------------------------- 600

Q56T -------------------------------------------------- 600

G63T -------------------------------------------------- 600

R67S -------------------------------------------------- 600

T90A -------------------------------------------------- 600

F143T -------------------------------------------------- 600

F143S -------------------------------------------------- 600

W169S -------------------------------------------------- 600

R67S/T90A -------------------------------------------------- 600

CTendo45 CTGGGTGCCGACAACCCGGACGTCTCTTTCAAGCAGGTGGCTTGCCCGGC 650

L47T -------------------------------------------------- 650

Q56T -------------------------------------------------- 650

G63T -------------------------------------------------- 650

R67S -------------------------------------------------- 650

T90A -------------------------------------------------- 650

F143T -------------------------------------------------- 650

F143S -------------------------------------------------- 650

W169S -------------------------------------------------- 650

R67S/T90A -------------------------------------------------- 650

CTendo45 AGCCATCACGGCCAAGAGCAAGTGCGTGCGACAGCGGGATGTCATCGACC 700

L47T -------------------------------------------------- 700

Q56T -------------------------------------------------- 700

G63T -------------------------------------------------- 700

R67S -------------------------------------------------- 700

T90A -------------------------------------------------- 700

F143T -------------------------------------------------- 700

F143S -------------------------------------------------- 700

W169S -------------------------------------------------- 700

R67S/T90A -------------------------------------------------- 700

CTendo45 AGACGCCGACTGGACCGGAGATTGTCCCGACTTGGACTCCCTAA 744

L47T -------------------------------------------- 744

Q56T -------------------------------------------- 744

G63T -------------------------------------------- 744

R67S -------------------------------------------- 744

T90A -------------------------------------------- 744

F143T -------------------------------------------- 744

F143S -------------------------------------------- 744

W169S -------------------------------------------- 744

R67S/T90A -------------------------------------------- 744

**Fig. S2.** Translated amino acids sequences of CTendo45 and designed mutants

CTendo45 MHLSQLALPLLLAAGAHAQGAQGTGRTTRYWDCCKPSCAWPGKSNALQPV 50

L47T ----------------------------------------------t--- 50

Q56T -------------------------------------------------- 50

G63T -------------------------------------------------- 50

R67S -------------------------------------------------- 50

T90A -------------------------------------------------- 50

F143T -------------------------------------------------- 50

F143S -------------------------------------------------- 50

W169S -------------------------------------------------- 50

R67S/T90A -------------------------------------------------- 50

CTendo45 QTCNAQDQPLNDGGNTRSGCDSGGSAFMCSNQSPWALNETLSYGWAAVRI 100

L47T -------------------------------------------------- 100

Q56T -----t-------------------------------------------- 100

G63T ------------t------------------------------------- 100

R67S ----------------s--------------------------------- 100

T90A ---------------------------------------a---------- 100

F143T -------------------------------------------------- 100

F143S -------------------------------------------------- 100

W169S -------------------------------------------------- 100

R67S/T90A ----------------s----------------------a---------- 100

CTendo45 AGQSEFNWCCACYELTFTSGPVAGKKMIVQATNTGGDLGSNHFDIAIPGG 150

L47T -------------------------------------------------- 150

Q56T -------------------------------------------------- 150

G63T -------------------------------------------------- 150

R67S -------------------------------------------------- 150

T90A -------------------------------------------------- 150

F143T ------------------------------------------t------- 150

F143S ------------------------------------------s------- 150

W169S -------------------------------------------------- 150

R67S/T90A -------------------------------------------------- 150

CTendo45 GVGIFNACTQQYGAPPNGWGERYGGIRSRSECDSFPEALKAGCYWRFDWF 200

L47T -------------------------------------------------- 200

Q56T -------------------------------------------------- 200

G63T -------------------------------------------------- 200

R67S -------------------------------------------------- 200

T90A -------------------------------------------------- 200

F143T -------------------------------------------------- 200

F143S -------------------------------------------------- 200

W169S ------------------s------------------------------- 200

R67S/T90A -------------------------------------------------- 200

CTendo45 MHLSQLALPLLLAAGAHAQGAQGTGRTTRYWDCCKPSCAWPGKSNAL 247

L47T ----------------------------------------------- 247

Q56T ----------------------------------------------- 247

G63T ----------------------------------------------- 247

R67S ----------------------------------------------- 247

T90A ----------------------------------------------- 247

F143T ----------------------------------------------- 247

F143S ----------------------------------------------- 247

W169S ----------------------------------------------- 247

R67S/T90A ----------------------------------------------- 247

**Fig. S3.** SDS-PAGE analysis of purified recombinant enzymes. Lane M, molecular mass markers; lane wt, the wild-type CTendo45; lane 1-3, R67S, T90A and R67S/T90A

M wt 1 2 3

kDa


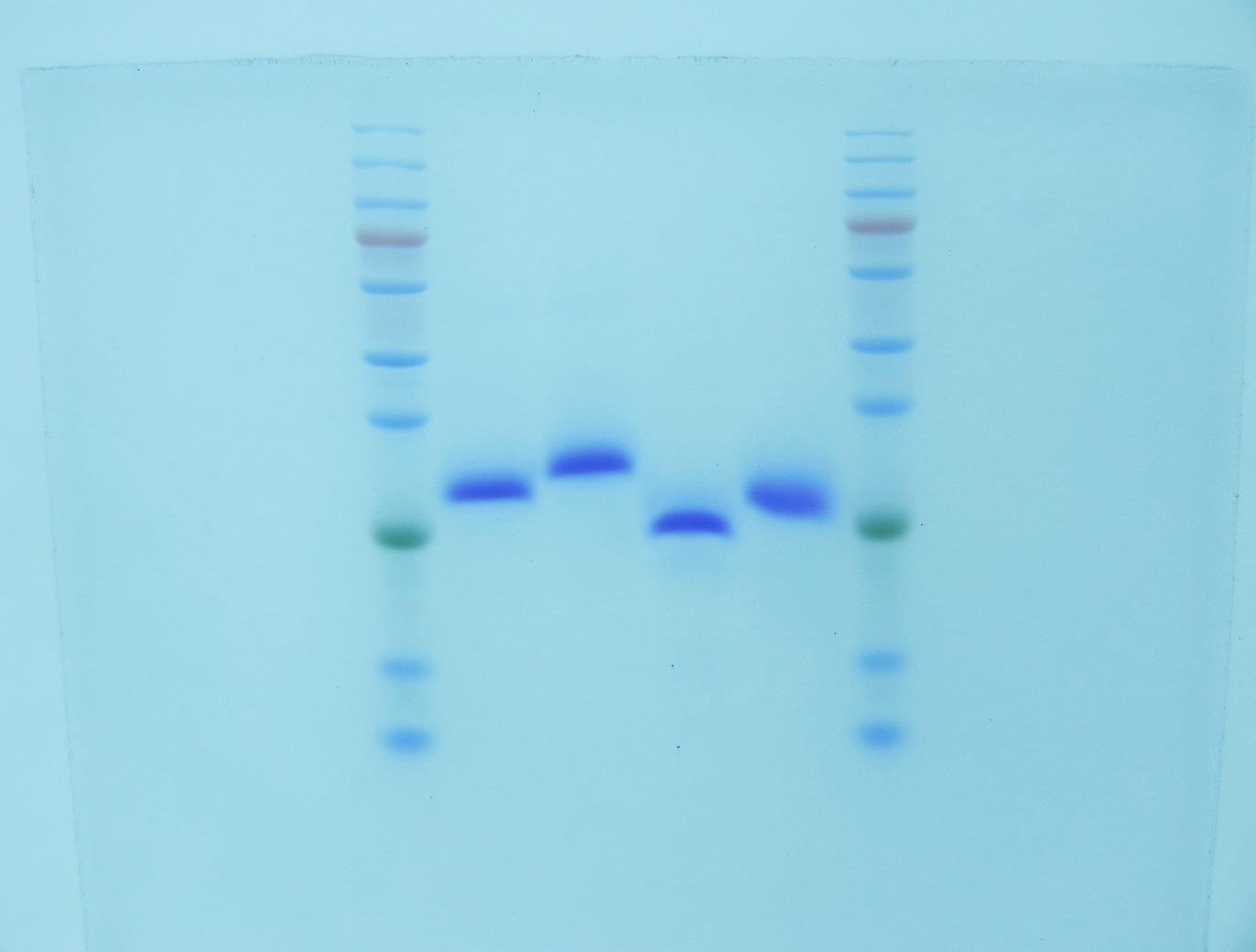


75

180

140

100

10

15

38

26

60

45

**Fig. S4.** Mass spectrum of tryptic digest of CTendo45 and its mutants. **a** The wild-type CTendo45. **b** L47T. **c** Q56T. **d** R67S. **e** T90A. **f** R67S/T90A. The molecular mass of peptide containing intrinsic N-glycosylation motif (N88-E89-T90) was noted by solid black box, and the additive N-glycosylation motif was remarked by solid red box. The black dotted box denotes the peptide fragments containing unglycosylated N88-E89-A90 motif. All these useful mass values and charge numbers were underlined

(a)


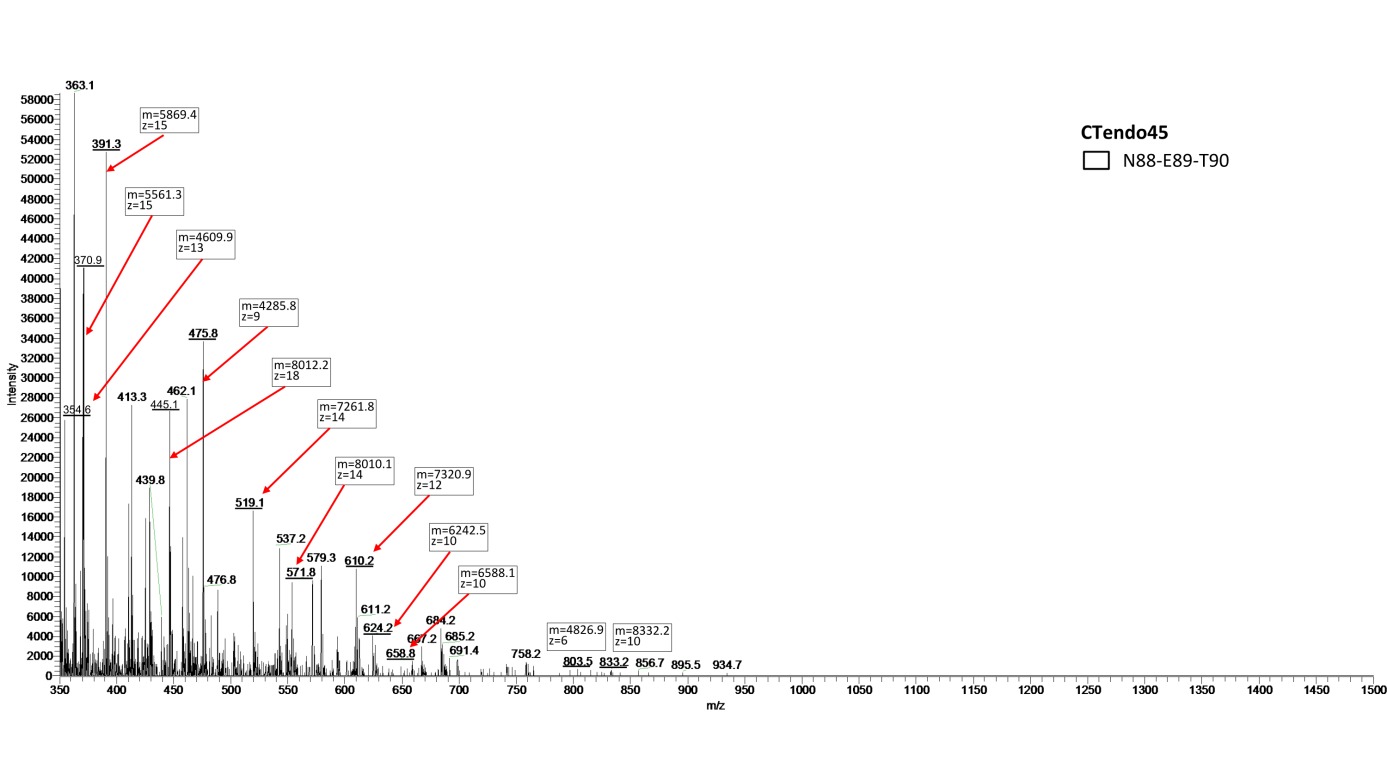

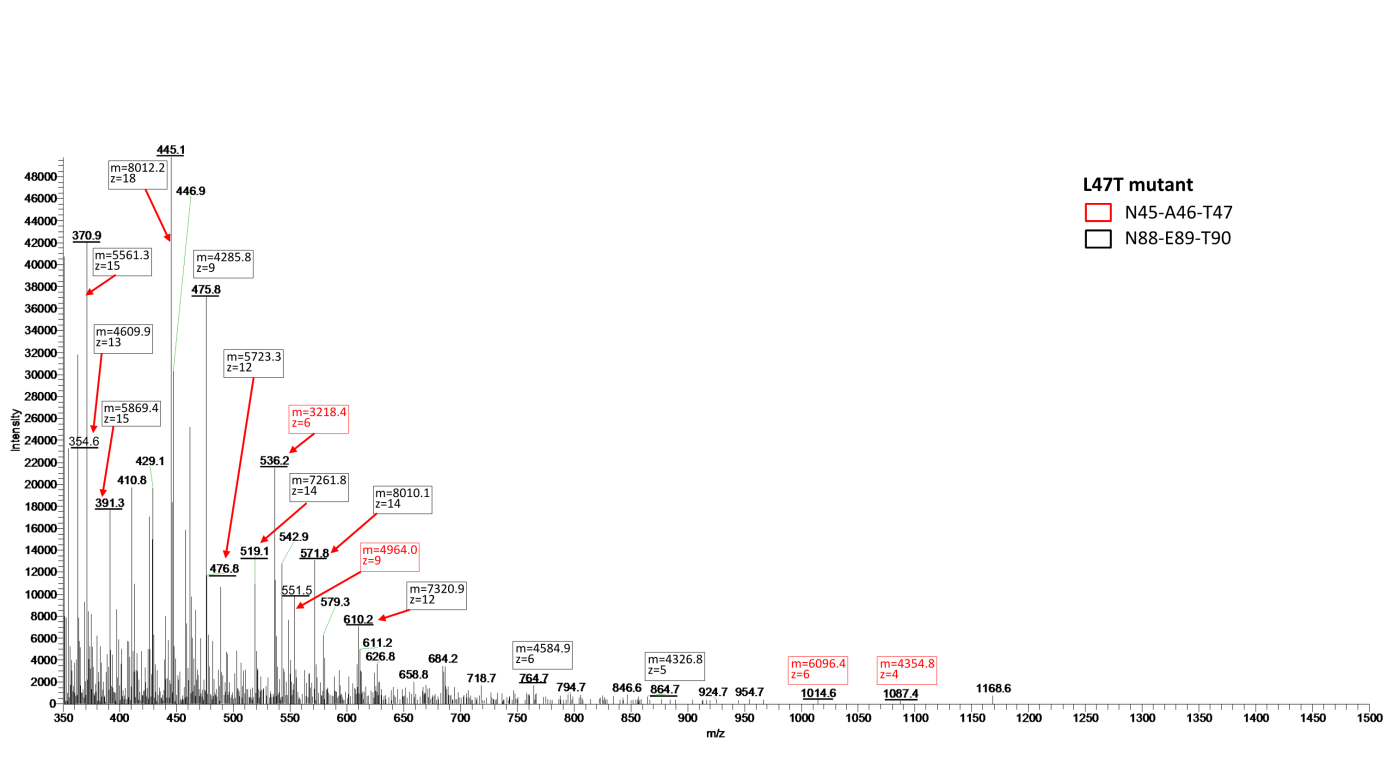


(c)

(b)


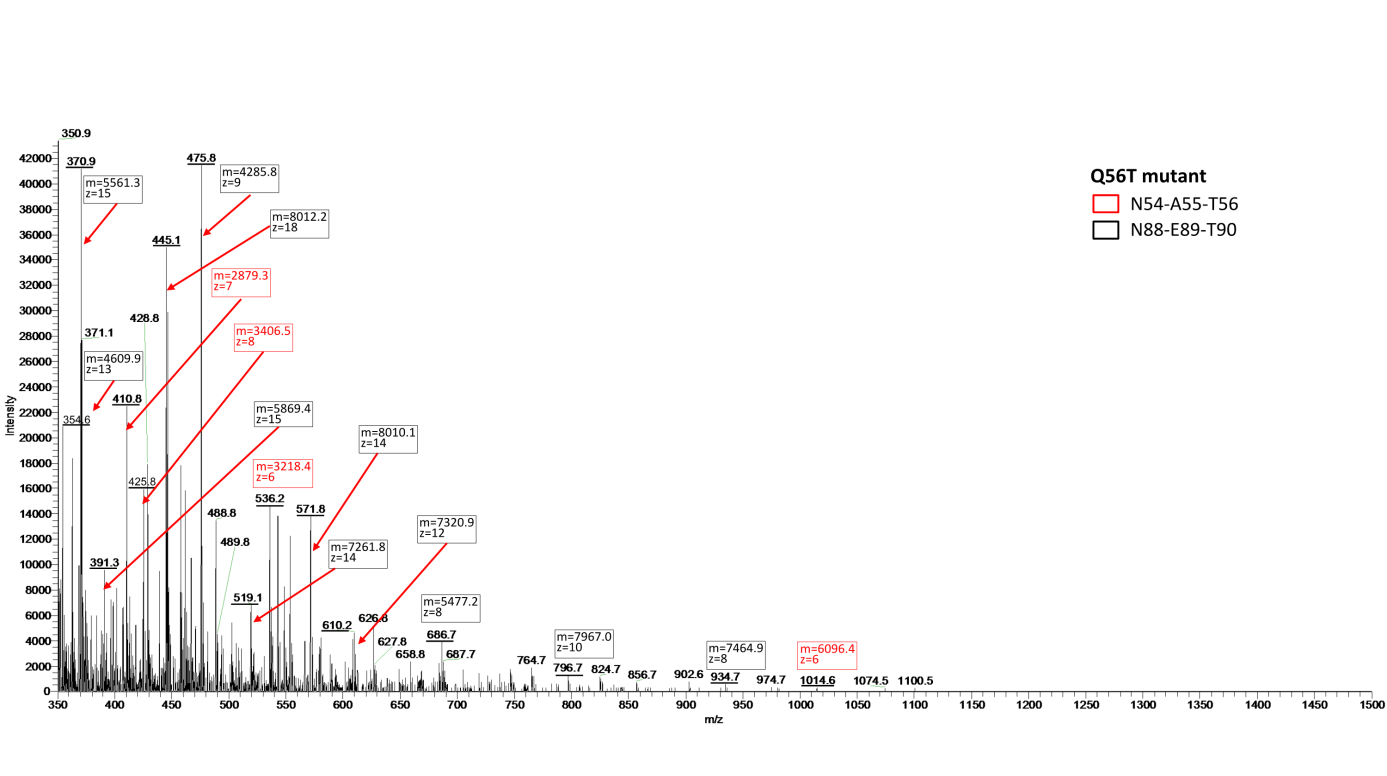


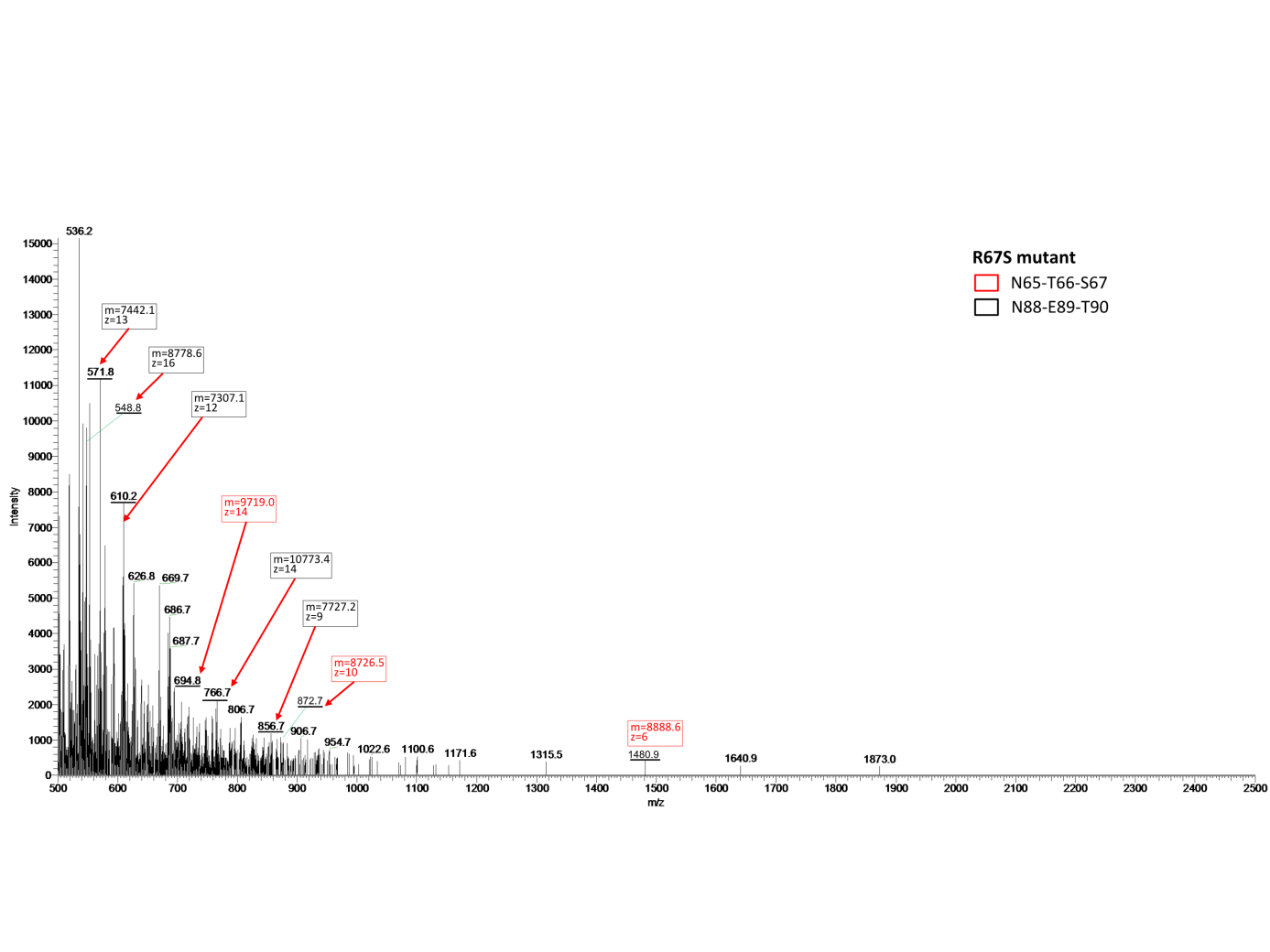


(e)

(d)


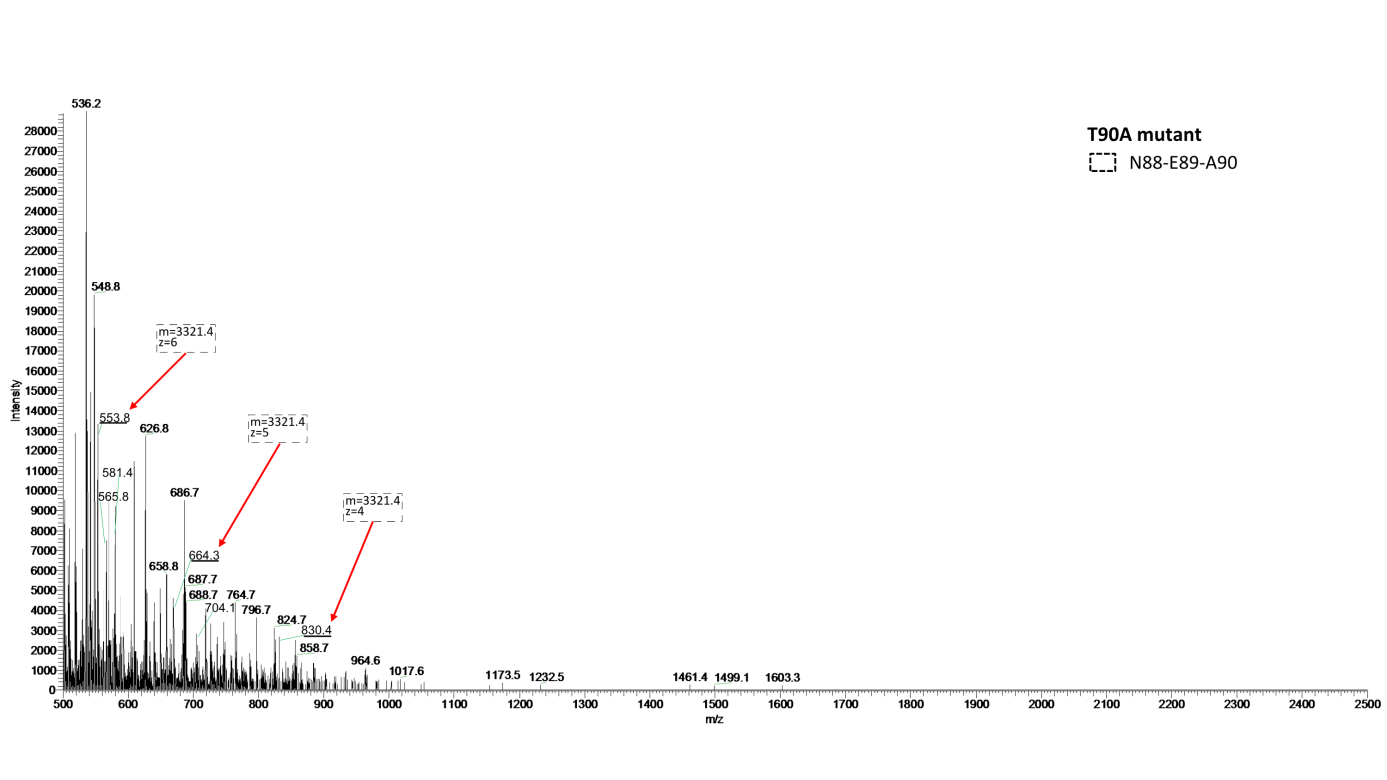


(f)


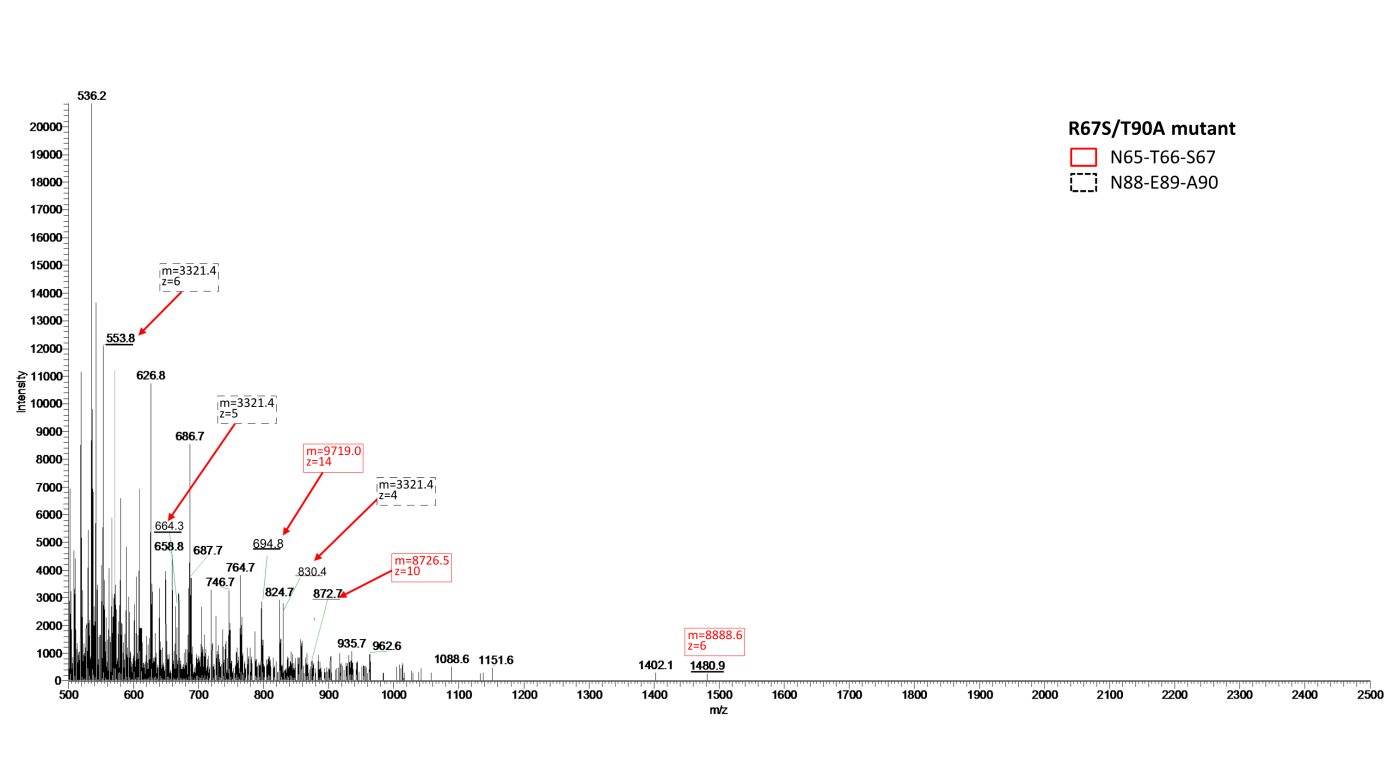


**Fig. S5.** Enzymatic properties of wild-type CTendo45 and its mutants against β-D-glucan. **a** The optimal reaction pH. **b** The optimal reaction temperature. **c** Thermostability


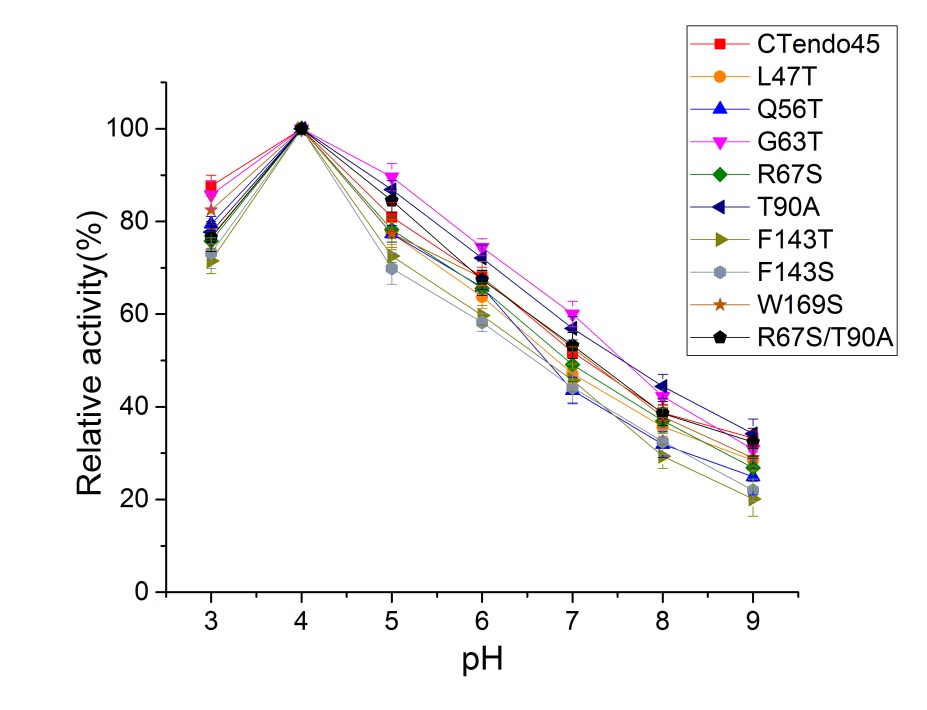


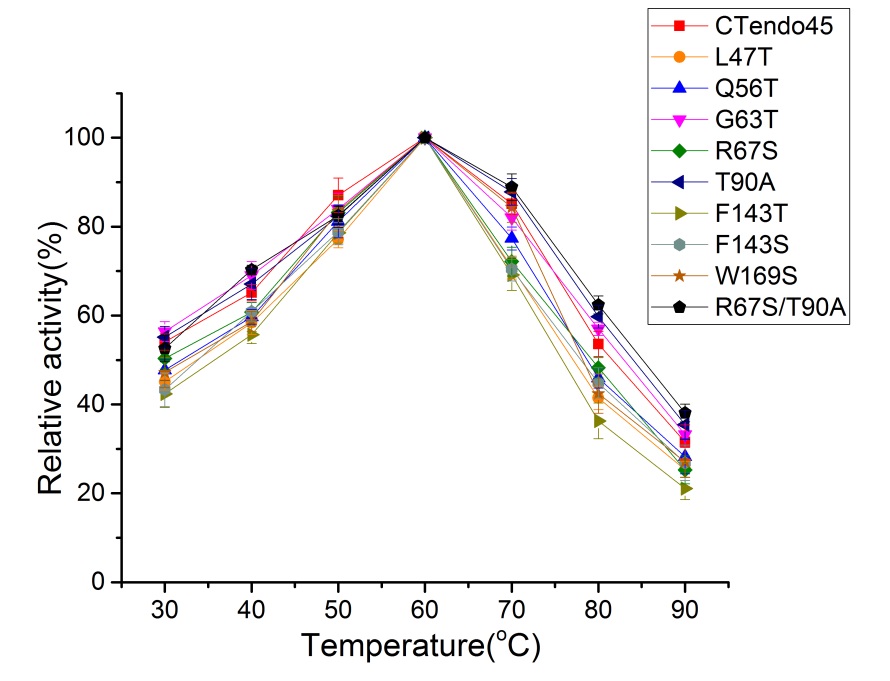


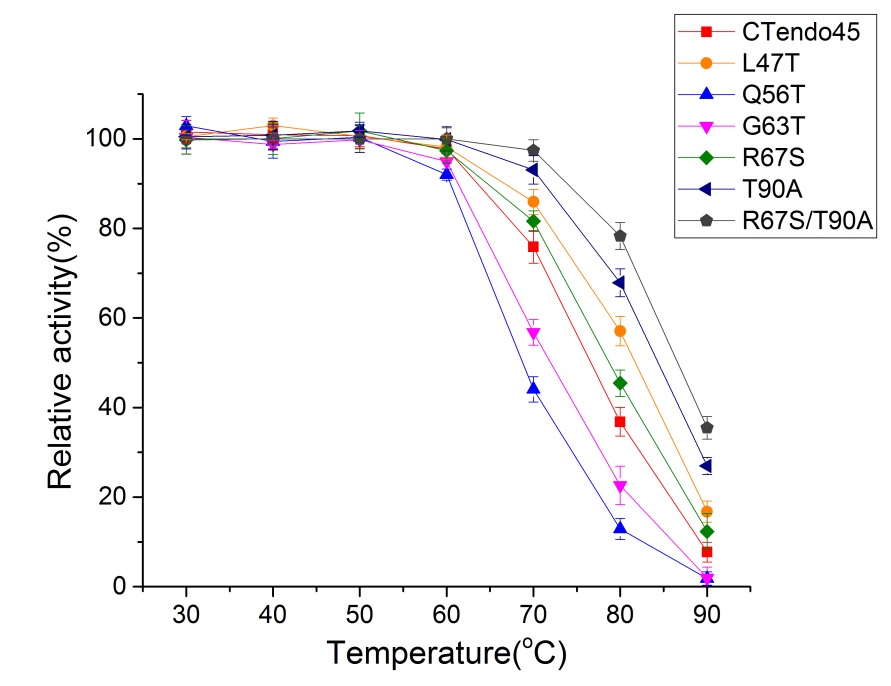


**Fig. S6.** Characterization of non-glycosylated versions of CTendo45. Heterologous expression of CTendo45 was performed using *Escherichia coli*. The *E. coli* str*ain* BL21 (DE3) (Invitrogen, Carlsbad, CA, USA) and the pEASY-Blunt E1 expression vector (TransGen Biotech, Beijing, China) were used for protein expression. After purification by Ni^2+^ affinity chromatography, the purified recombinant enzyme, termed as Ec45, was obtained. Besides, CTendo45 was treated with PNGase F and the generated non-glycosylated form was termed as CTPNG. Then, the catalytic activity and thermostability of Ec45 and CTPNG were detected using 1% (w/v) CMC-Na as a substrate. The reaction was conducted at 60°C, pH 4.0 for 30 min. Thermostability was determined by detecting the residual activities after the enzymes were pre-incubated at 30-90°C for 200 min **a** SDS-PAGE analysis. **b** Hydrolysis activity. **c** Thermostability

(b)

(a)


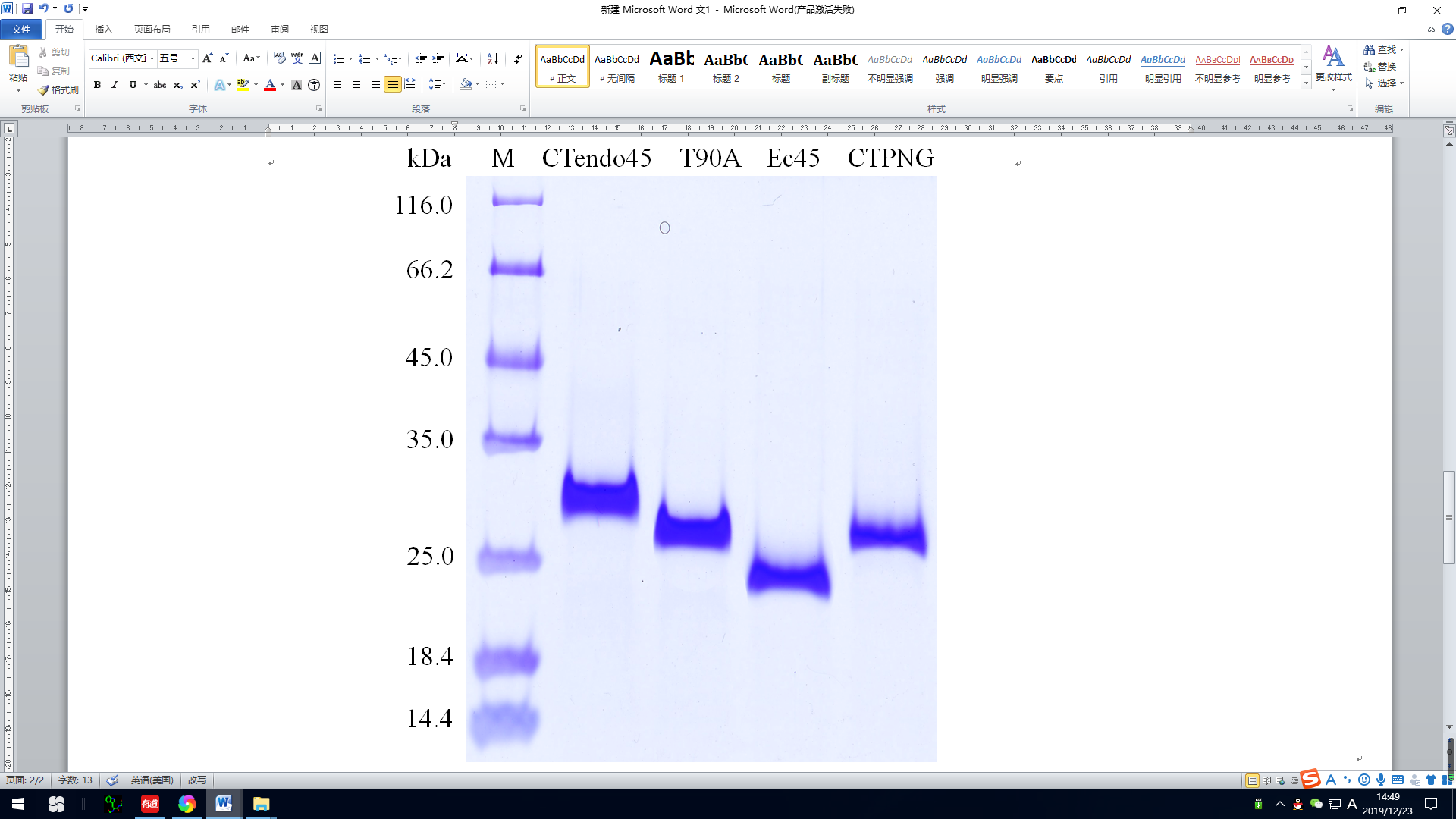

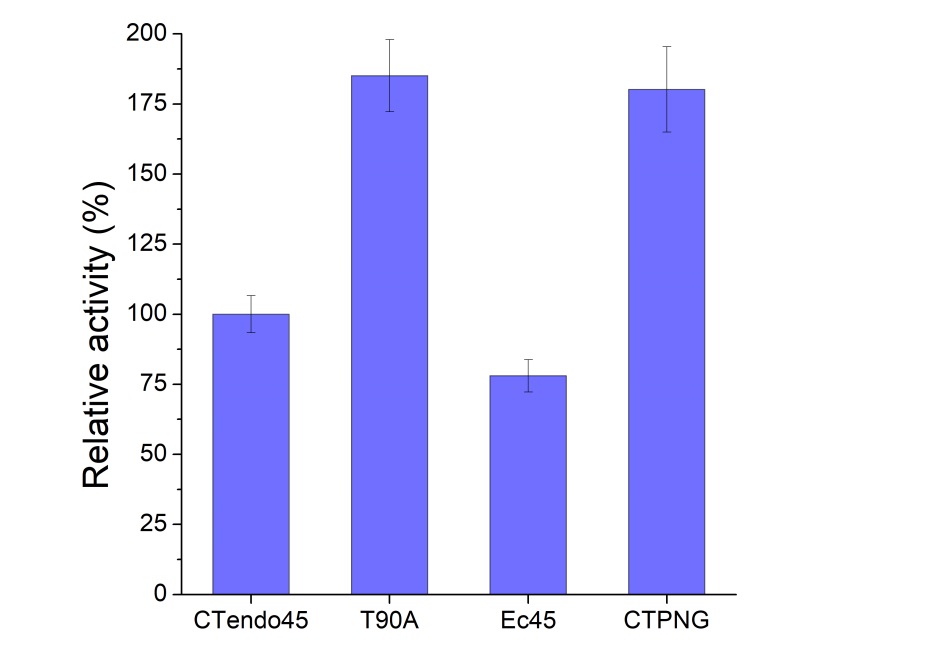


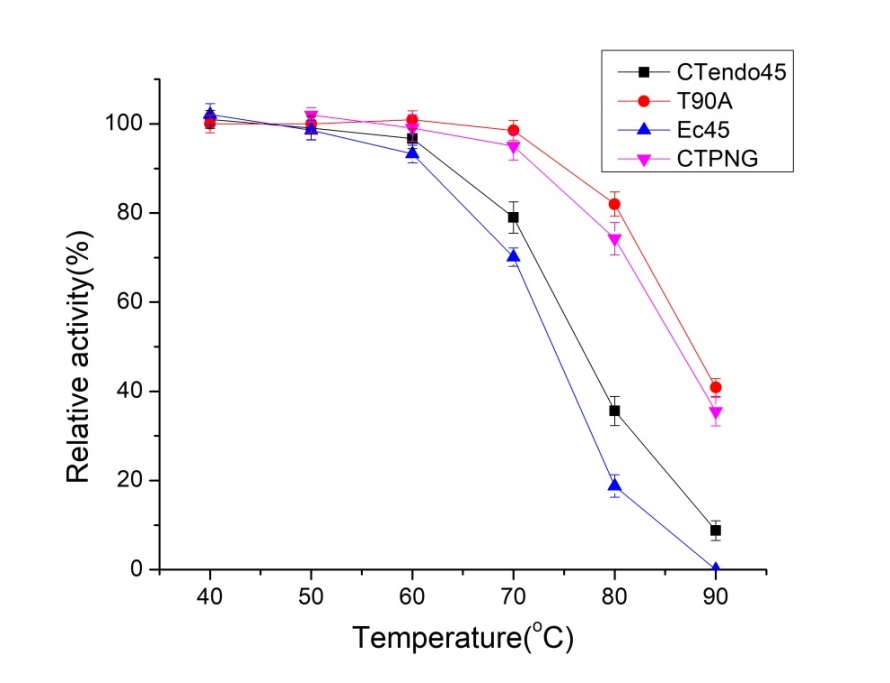


(c)
